# Early amphibians evolved distinct vertebrae for habitat invasions

**DOI:** 10.1371/journal.pone.0251983

**Published:** 2021-06-09

**Authors:** Aja Mia Carter, S. Tonia Hsieh, Peter Dodson, Lauren Sallan

**Affiliations:** 1 Department of Earth and Environmental Sciences, University of Pennsylvania, Philadelphia, PA, United States of America; 2 Department of Biology, Temple University, Philadelphia, PA, United States of America; 3 Department of Biomedical Sciences, School of Veterinary Medicine, University of Pennsylvania, Philadelphia, PA, United States of America; 4 Department of Biology, University of Pennsylvania, Philadelphia, PA, United States of America; Universidade de Sao Paulo, BRAZIL

## Abstract

Living tetrapods owe their existence to a critical moment 360–340 million years ago when their ancestors walked on land. Vertebrae are central to locomotion, yet systematic testing of correlations between vertebral form and terrestriality and subsequent reinvasions of aquatic habitats is lacking, obscuring our understanding of movement capabilities in early tetrapods. Here, we quantified vertebral shape across a diverse group of Paleozoic amphibians (Temnospondyli) encompassing different habitats and nearly the full range of early tetrapod vertebral shapes. We demonstrate that temnospondyls were likely ancestrally terrestrial and had several early reinvasions of aquatic habitats. We find a greater diversity in temnospondyl vertebrae than previously known. We also overturn long-held hypotheses centered on weight-bearing, showing that neural arch features, including muscle attachment, were plastic across the water-land divide and do not provide a clear signal of habitat preferences. In contrast, intercentra traits were critical, with temnospondyls repeatedly converging on distinct forms in terrestrial and aquatic taxa, with little overlap between. Through our geometric morphometric study, we have been able to document associations between vertebral shape and environmental preferences in Paleozoic tetrapods and to reveal morphological constraints imposed by vertebrae to locomotion, independent of ancestry.

## Introduction

Paleozoic tetrapods were the first vertebrates to invade and diversify on land. The biological changes necessary for this invasion have been studied extensively, mainly focusing on the evolution of robust limb girdles, respiratory physiology and mechanics, and the development of the urinary system [1, 2]. The vertebral column is also vital for supporting weight on land, and in aquatic reinvasions was essential for swimming [3–8]. Yet, most studies of the relationship between spinal morphology and terrestriality in Paleozoic tetrapods have been qualitative, with habitat preferences inferred from general form [9]. Generally, vertebral morphologies that reduce flexibility in axial torsion and dorsoventral flexion are believed to indicate terrestrial lifestyles [1, 2, 10–15]. Early tetrapods are typically inferred to have decreased flexibility if they have broad neural spines with robust horizontal zygapophyses and low vertebral counts [1, 2, 10–15]. Conversely, aquatic early tetrapods have been described as those that bear vertebrae with caudally sloping and displaced neural spines, with ventrally sloping prezygapophyses [10, 16].

The classic qualitative descriptions of terrestrial versus aquatic vertebral forms inspired our quantitative investigation of the changes in the functional morphology of a select clade of Paleozoic tetrapods, the temnospondyls [17–19]. The few previous quantitative efforts usually focused on single early tetrapod species or a handful of living amphibious amniotes with superficially similar vertebrae [9, 12, 13, 15, 20–23]. These studies lacked both phylogenetic context and explicit testing of the association between habitat preference and vertebral form. Temnospondyls (stem-Lissamphibia:frogs, caecilians, salamanders; [24]) represent a significant segment of overall Paleozoic tetrapod vertebral diversity. They are also diverse in terms of both species counts and life modes [25]. This group also has well-characterized phylogenetic relationships allowing for the use of comparative phylogenetic methods [25]. These factors make them an appropriate study group for extensive macroevolutionary investigations of morphological correlates of habitat [25].

The amphibious biology of Temnospondyli is well established because aquatic larval-to-terrestrial adult growth series exist for multiple species [26–28]. Temnospondyls are found in depositional environments that range from arid upland to entirely marine [29–31]. This group exhibited a range of adult body lengths from five centimeters to six meters [32]. Despite the plethora of information on temnospondyl life-styles, previous studies to elucidate vertebral function have been limited by various factors. For example, some previous functional models were limited in taxonomic scope, e.g., one to three species as individuals [12, 15, 20, 33], used questionable material properties (e.g., a garden hose as notochord material [12]); or presented little to no quantitative analyses [13, 23, 34]. Comparative, quantitative studies of vertebral morphology will provide more insight into morphological patterns critical for subsequent reinvasions of aquatic environments.

### Temnospondyl vertebral forms

Combinations of embryological [35, 36] and paleontological [37, 38] descriptions aided early workers in categorizing early tetrapod vertebral groups based on osteological elements present in a single vertebral unit [39, 40]. For the remainder of this study, "vertebral types" refers to these compositionally defined types. In stem tetrapods and stem lissamphibians, rhachitomous (1a), reverse rhachitomous (1b), stereospondylous (1c), and plagiosaurid (1d), vertebral types are represented [10, 11]. For additional early tetrapod vertebral morphologies outside the scope of this paper we recommend [17] for further review.

**Fig 1:**
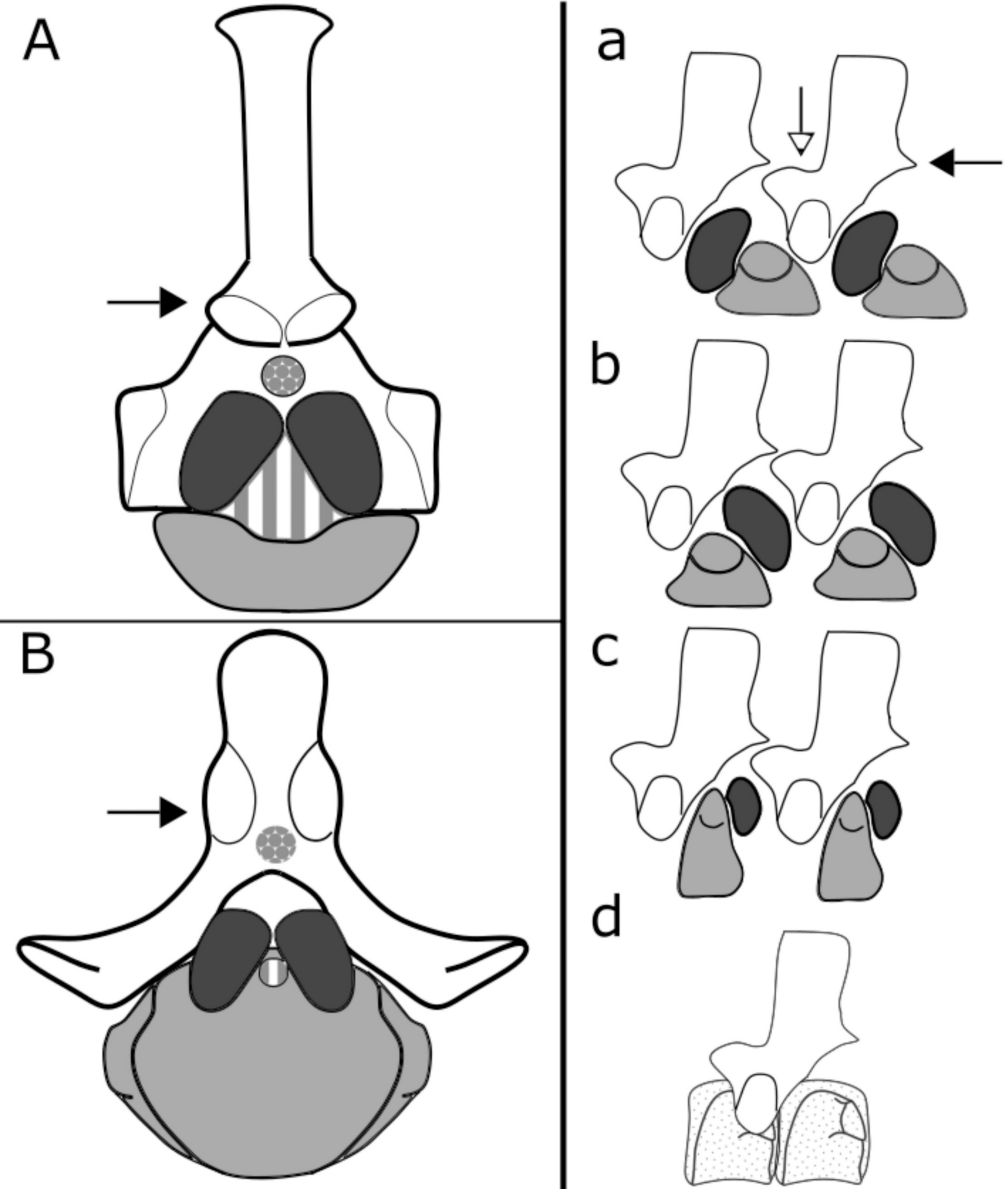
Temnospondyl vertebral types. Neural arches in white, pleurocentra in dark gray, intercentra in light gray, spinal canal in packed gray circles, notocanal in striped bars. **Left column**; rhachitomous vertebra from *Eryops megacephalus* (FMNH 117) (A) and stereospondylous vertebra from *Mastodonosaurus giganteus* (AMNH 2994) (B), vertebrae in caudal view. **Right column:** variations on rhachitomous vertebrae seen in Temnospondyli; (a) Rhachitomous; (b) Reverse Rhachitomous; (c) Stereospondylous; (d) Plagiosaurid. White arrows point to prezygapophyses, black arrows point to postzygapophyses.

Temnospondyls broadly have three major vertebral types: rhachitomous, stereospondylous, and the plagiosaurid (Fig 1A, 1B and 1b–1e) [17]. These can all be considered variations of rhachitomous vertebrae in terms of composition [17]. Rhachitomous vertebrae consist of a cranioventral crescentic ring (the intercentrum), paired caudodorsal pleurocentra, and the neural arch dorsally (Fig 1A, 1B and 1b). In more derived temnospondyls, the pleurocentra can exhibit severe reduction or absence (i.e., stereospondylous condition, Fig 1c). The most modified form within temnospondyls is the plagiosaurid condition, in which two enlarged centra share one neural arch. The homology, whether the two central elements are two intercentra, subequal enlarged intercentra and pleurocentra, or complete fusion of the intercentra, is still debated [16, 41–43]. In recent years the rhachitomous vertebral form, prominent in temnospondyls and early stem tetrapods, has been considered ancestral to all tetrapod vertebral forms (in contrast to Romer’s early ancestral embolomere hypothesis [9, 16, 40]) and thus to make up the remainder of diversity in vertebral form and the bulk of species diversity in the Carboniferous and Permian [17, 25, 40].

We focused on neural arches and intercentra because of the availability of material, knowledge of functional morphology from modern analogs, and because they have been hypothesized to have a major role in terrestrial locomotion and weight-bearing. We did not consider pleurocentra due to debates over homology and inconsistent distribution across Temnospondyli [16]. Below, we discuss predictions for neural arch and intercentum morphology in different environments based on the prior assumptions of an association between form and a need for weight-bearing capacity on land.

### Neural arches

Neural arches consist of the neural spine (also called the spinous process), the spinal canal, and in tetrapods and some high-speed swimming fishes, articular facets (including pre- and postzygapophyses) and laminae (Fig 1) [44]. Zygapophyses on neural arches influence the range of motion in intervertebral joints and demonstrate which planes of motion (axial, lateral, dorsoventral) are restricted [8, 15, 34–37, 45–48]. Therefore, specific orientations of these articular facets have been used as indicators of terrestrial or aquatic forms of locomotion in extant and fossil organisms [14]. In particular, terrestrial temnospondyls would be expected to exhibit prezygapophyseal morphologies that resist ventral shear, which would support the animal on land, by reducing trunk sag while locomoting [13, 15, 21]. Ventrally-angled articular facets are not considered to be beneficial in resisting trunk sag [15, 45, 49]. Generally, aquatic temnospondyls are expected to exhibit more distinct differences in zygapophyseal orientation, suggesting regional flexibility to a greater extent than has been reported in terrestrial temnospondyls [16].

In addition to pre- and postzygapophyses, neural spine morphology indicates changes in origin and insertion of axial myology, and thus changes in neural spine form may be reflective of changes in locomotion [50–53]. Lateral axial bending characterizes terrestrial locomotion in extant amphibians and is assumed to have facilitated walking in stem lissamphibians [1, 7, 54, 55]. In extant lissamphibians, muscles attached to the neural spine control lateral bending (dorsalis trunci) and stabilize the intervertebral joints while the spine is bending (interspinalis) [7, 55, 56]. Comparative myology studies on extant salamanders (Caudata) have demonstrated that terrestrial taxa have larger dorsalis trunci muscles. Researchers have posited that this difference is due to dorsalis trunci muscles having a secondary role of preventing sagging and torsion during terrestrial walking [8, 54–56]. Additionally, terrestrial taxa require more stabilization of intervertebral joints and rely less on the vertebral column as a primary driver of locomotion [57]. Previous investigators demonstrated prolonged activation in the interspinalis muscle (local joint stabilization muscle) in terrestrial walking compared to aquatic forms of locomotion [8]. The dorsalis trunci and interspinalis muscles both insert on the neural spine, and changes to the spine are indicative of changes in muscle attachment area and mechanical torque.

Decreasing the space between neural arches by increasing the craniocaudal length of the neural spine relative to the centra would increase intervertebral rigidity by enclosing the interarticular space [47, 49, 53, 58]. Additionally, increasing dorsoventral height of the neural spine would increase attachment area for the interspinalis and dorsalis trunci muscle and lead to an increase in intervertebral rigidity and aid in preventing trunk tag. Lastly, neural spines closer to 90 degrees vertical indicate greater torque for the dorsalis trunci and interspinalis muscles.

### Intercentra

Centra in early and extant tetrapods are weight-bearing elements [13]. Previous biomechanical studies suggest that centra height, length and convexity are correlated to rigidity of intervertebral joints [3, 4, 46, 47, 49, 52, 59–61]. If we first assume similar intervertebral height and length, centra with greater convexity increase rotation at the joint by decreasing centrum-centrum contact [49]. If instead centra convexity is maintained and centra length is greater than centra height, intervertebral joints are increasingly rigid as compared to shorter centra, which are associated with intervertebral flexibility, particularly lateral flexion [46, 47, 49]. In this study, we focus on intercentrum morphology, but we note that the changes in total vertebral numbers are also related to overall spinal flexibility [4, 62, 63]. Indeed, an increase in the number of overall vertebral units could indicate an increase in spinal flexibility, all else being equal [10, 64].

Terrestrial taxa are assumed to require spines which are more rigid along the dorsoventral axis, with some lateral flexibility for locomotion on land, with motion limited by the intervertebral joints [1, 12, 65]. As a result, we hypothesize terrestrial temnospondyls would have intervertebral morphologies that increase intervertebral rigidity compared to aquatic taxa. We hypothesize terrestrial temnospondyls would have craniocaudally elongated neural spines, with prezygapophyses that have a greater vertical orientation than aquatic taxa and intercentra forms craniocaudally longer than those of aquatic temnospondyls. Here, we use geometric morphometrics and a Bayesian phylogenetic approach to examine the vertebral form and terrestriality using temnospondyls as a focal group.

## Methods

### Data collection

We reconstructed an evolutionary tree for temnospondyls from the Ruta et al. [66] supertree of 172 taxa using Newick trees in nexus format to make the tree readable in R for our comparative phylogenetic analyses. We obtained the stratigraphic stages for taxon occurrences from Ruta et al. [66] and used the geological units to determine maximum and minimum ages for our terminal taxa. We generated a single date for each species within that range, and time-scaled our tree under the "equal" method in *Strap* [67] with a minimum branch length of 1 million years. We pruned our time-scaled tree to match the sample sizes for intercentra and neural arches to be used in our phylogenetic ANOVAs on shape, using the code and procedures from Sallan et al., 2018 [68].

We compiled habitat information for temnospondyls from the literature and existing databases, including inferences from functional and histological studies of limbs, depositional habitats noted in descriptions, finite element analysis studies, and environmental assignments for specific beds in the Paleobiology Database (PBDB; Supplemental information). All types of studies were weighted equally in the ecology database (i.e., we did not favor experimental studies only or morphological studies only). This allowed us to generate the sizeable ecological dataset for this study and avoid subjectivity introduced by discretizing and weighting data sources. Many well-cited temnospondyl studies are anatomical or morphological in nature. Such studies do not produce standard deviations or means to conduct any form of statistical meta-analysis but are nevertheless informative [69, 70]. Designations from the same authors for the same taxon were only counted once, and only the most recent publication were added to our dataset. Primary sources, i.e., not conclusions taken from secondary sources, were used for the final environmental data. We considered these to be independent sources for our first dataset. As more individuals are discovered and more empirical techniques are available for study, previous ecological hypotheses can be overturned. We selected ecological data from the most recent publication, regardless of author, for a given taxon to address potential changes over research history concerning ecology. We treated these two collections of ecological data as two separate data sets, a “consensus” data set and a “most-recent” data set. The habitat data were used to categorize each temnospondyl taxon within our trees as aquatic, semi-aquatic, or terrestrial. Each discrete categorization was based on the environment in which the taxon would have spent most of its adult life, barring reproduction, as all temnospondyls are believed to have been amphibious in reproduction [26–28]. For our Bayesian analyses, we assigned prior probabilities for occurrence of each taxon in each habitat and rounded these up to the first significant digit [68]. For example, eight sources stated that *Eryops* was terrestrial (8/9 sources) and one source (1/9 source) stated *Eryops* to be semiaquatic (Supplemental information); thus, our phylogenetic analyses used a prior of 0.9 for terrestrial and 0.1 for semiaquatic, while it was assigned as terrestrial in our morphospaces. The most probable habitat alone was used as the assumed state for constructing convex hulls and calculating disparity in our geometric morphometric study in our “ecology consensus data” set. We also conducted separate analyses using the ecological classification from the most-recent publication regarding each taxon (S2-S4 Tables in S1 File).

To generate our morphological dataset, we collected images of trunk intercentra and neural arches from the literature, and photos from museum specimens with well-preserved lateral views (museum speciemsn only displayed in Table 1). We assumed all vertebral elements came from adult specimen as all elements were heavily ossified [14, 71–73]. To collect only trunk vertebrae, in the case of museum specimens, we identified trunk intercentra with parapophyses for rib atachments and a lack of chevron facets [thus eliminating cervical, sacral and caudal vertebrae]. We selected trunk vertebrae because the animal would have had less direct support from the limbs in this region of the body. Any functional forces that might affect vertebral morphology would be isolated in this region of the body [13]. For museum photographs, specimens with minimal deformation were selected. We photographed vertebral elements in lateral view with the vertebral element in the middle of the picture frame to avoid issues of parallax. In addition, we also collected data from catalogued specimen reconstructions in the literature (S2-S4 Tables in S1 File). In total, we collected data on 32 intercentra and 29 neural arches (S2-S4 Tables in S1 File) of the presacral series. Twenty one species had both intercentra and neural arches accounted for (S2 Table in S1 File), eleven only had neural arches (S3 Table in S1 File), and twelve species only had intercentra (S4 Table in S1 File). These data sets were not combined. Different numbers of landmarks were required to accurately describe the shapes of both the intercentra and neural arches (S1 Table and, S1 Fig in S1 File). As there are different numbers of landmarks they could not be combined for partial least squares, integration, or modularity analyses [74]. The vertebrae we selected include representatives of every major temnospondyl clade as described by the supertree, as well as every major vertebral type in the presacral series seen within Temnospondyli [17–19]. The species varied in total body length (0.5–6 meters), depositional environment (marine–terrestrial environments), and temporal range (Carboniferous-Cretaceous).

**Table 1:** Museums used in this study.

	TAXON	SPECIMEN NUMBER
**NEURAL ARCHES**		
	*Archegosaurus dechani*	^a^YPM 9621
	*Aspidosaurus glascocki*	^b^AMNH 23412
	*Broiliellus novoamericanus*	^c^FMNH 1041
	*Dissorophus multicinctus*	^d^USNM 15555
	*Eryops megacephalus*	^c^FMNH 745
	*Koskinonodon perfecta*	^a^YPM 60249
	*Metoposaurus giganteus*	^b^AMNH 3097
	*Platyhystrix rugosus*	^e^UCMP 33437
	*Trimerorhachis insignis*	^e^UCMP 105157
**INTERCENTRA**		
	*Batrchosuchus browni*	^e^UCMP 42856
	*Cylcotosaurus roboustus*	^e^UCMP V3957
	*Wellesaurus peabodyi*	^e^UCMP 56110
	*Stenotosaurus semicalusus*	^e^UCMP 56108
	*Thanbanchuia oomie*	^c^FMNH 1029/5

For additional information on taxon used in this study refer to S2-S4 Tables in S1 File

^a^Yale Peabody Museum (YPM), New Haven, Connecticut, United States of America.

^b^American Museum of Natural History (AMNH), New York, New York, United States of

cAmerica. Field Museum of Natural History (FMNH), Chigago, Illinois, United States of America.

^d^Smithsonian National Museum of Natural History (USNM) City, State, United States of America.

^e^University of California Museum of Paleontology (UCMP), Berkeley, California, United States of America.

### Quantification and statistical analysis

#### Ancestral state reconstruction

We fit Bayesian threshold models to sampled habitat data for 172 out of 180 accepted temnospondyl genera within a phylogenetic context. The threshold model, as implemented in AncThresh in the R package Phytools [75] allows us to reconstruct discrete character changes by modeling “liability” [76–78], an underlying continuous character that follows a normal distribution of change. Liability is assumed to represent evolutionary cost, or the amount of morphological and physiological change, required to shift between habitats. One model parameter estimated in each generation by AncThresh is the threshold value of liability required to change between observed states given the topology of the tree. We ordered our life mode states in three possible configurations: 1) terrestrial to semiaquatic to aquatic; 2) aquatic to semiaquatic to terrestrial; 3) terrestrial to aquatic to semiaquatic. A lack of identifiably semiaquatic temnospondyls early in the fossil record precluded the use of a semiaquatic-first sequence, while the terrestrial to aquatic option was chosen to reflect potential paedomorphosis. AncThresh holds the threshold liability between the first two states constant at 0 [75, 76]. We tested the following combinations of the first two states, terrestrial to semiaquatic, aquatic to semiaquatic, and terrestrial to aquatic.

We ran each AncThresh analysis for 10 million generations using our habitat priors and our total phylogeny, applying the available Brownian Motion (BM), Ornstein-Uhlenbeck (OU), and Pagel’s Lambda (LB) models, with the first 1 million generations excluded as "burn-in” [68, 76]. We then used the Deviance Information Criterion to calculate DIC weights for model selection [68, 76]. The life-mode order with the lowest DIC value was selected, similarly to Revell (Supplemental information) [76]. We pruned our resulting trees to the level of major groups for clarity in Fig 4 and plotted to time in Strap [67]. The raw result trees are available in S5 Fig in S1 File. For a more complete explanation of these methods, see Sallan et al. [68].

#### Geometric morphometrics

Fossil centra are often incomplete, warped, disarticulated, and/or buried in matrix, rendering it difficult to obtain three dimensional reconstructions, particularly for many temnospondyls. Fortunately, neural arches (barring the transverse processes) and intercentra are relatively flat in transverse sections and have already been imaged in lateral view for many species. Thus, we opted to use 2D geometric morphometric techniques to maximize our sample size. As movement is assumed to be lateral, we can assume minimal loss of functionally-relevant information [79, 80]. All landmarks were digitized using *Geomorph* and all subsequent analyses were completed in *Geomorph*, *Phytools*, and *Geiger* [74, 75, 81]. We digitized a total of seventeen landmarks and nine semilandmarks to capture curves on the neural arches and in a separate analysis twelve landmarks and eight semilandmarks on the intercentra (S1 Table and; S1 Fig in S1 File). We used the function *LaSEC*, from the *Landmark Sampling Evaluation Curve* package [82] to confirm that the number of landmarks adequately described the shape variation among the vertebral elements (S2 Fig in S1 File). To account for shape differences as related to specimen type (museum photo, reconstruction, literature photo, literature drawing), we included them in the multivariate shape ANOVA. We then conducted a generalized Procrustes analysis and principal component analysis using *gpagen* and *plotTangentspace* in *Geomorph* to generate a morphospace [74]. This generated morphospace allows us to see the shape variation in intercentra and neural arches of temnospondyls. We generated two morphospaces per vertebral element, morphospaces with outliers (as calculated by the plot.Outliers function) and morphospaces without. We also produced backtransform morphospaces using custom code in the *Stereomorph* package. These plots allowed us to see variation in both neural arches and intercentra by plotting the distribution of morphologies along particular principal components [83]. We used both a scree-test and the Jolliffee cut-off (eigenvalues that proportions add to minimum 70% of the variance) [84, 85] (S3 Fig in S1 File). We then used statistically significant patterns (see below *Correlation Between Habitat and Shape)* to infer mechanical properties related to function on the morphospaces with no outliers. The generalized Procrustes analysis produces centroid sizes, and new coordinates in the shape space.

In vertebrates, there is an association between the surface area on the lateral side of the neural spine and lateral area of attachment of the dorsalis trunci and interspinalis musculature, and so we inferred relative muscle attachment area from the centroid sizes, or surface areas, in our general Procrustes analysis [57, 86]. We calculated shape disparity using the *morphol*.*disparity* function in the *Geomorph* package. This is a permuted and iterative procedure to handle our small sample size. Our morphological disparity test compared the Procrustes variances of shape and centroid sizes among our inferred habitat groups.

#### Correlation between habitat and shape

After checking for linearity (diagnostic plots in *procD*.*lm*) we used a factorial ANOVA to determine if vertebral shapes (morphology and potential muscle attachment sites) were correlated with habitats using the *procD*.*lm* function in *Geomorph* [74]. Our factorial ANOVA compared the means of the centroid sizes and Procrustes distances of the previously established a priori life-mode/habitat preference (aquatic, semiaquatic, and terrestrial) groups to the overall sample mean (α<0.05). To determine the degree to which muscle attachment, vertebral shape, and habitat were determined by ancestry, we conducted a factorial ANOVA on residuals that we then permuted across the neural arches and intercentra trees. We then calculated Bloomberg’s K, a value indicating phylogenetic signal [74]. Finally, we conducted a phylogenetic least squares (pgls) analysis in *Geomorph* to test for correlation between vertebral shape and environments independent of ancestry [74].

We tested for a correlation between life-mode and shape independent of phylogeny using *ThreshBayes* in the R package *Phytools*, which applies a Bayesian threshold model for discrete characters as above, with change simulated only under Brownian Motion [76–78]. This used our prior probabilities for lifemode, the transition sequence from our best-fit OU model (terrestrial-semiaquatic-aquatic) and the principal component (PC)1 scores from our morphospaces. As for our *AncThresh* analyses, we ran each analysis for 10 million generations with the first 1 million excluded for our "burn-in". *ThreshBayes* returns a most probable effect size (r) and a correlation coefficient (r2).

## Results

### Neural arch shape

**Fig 2:**
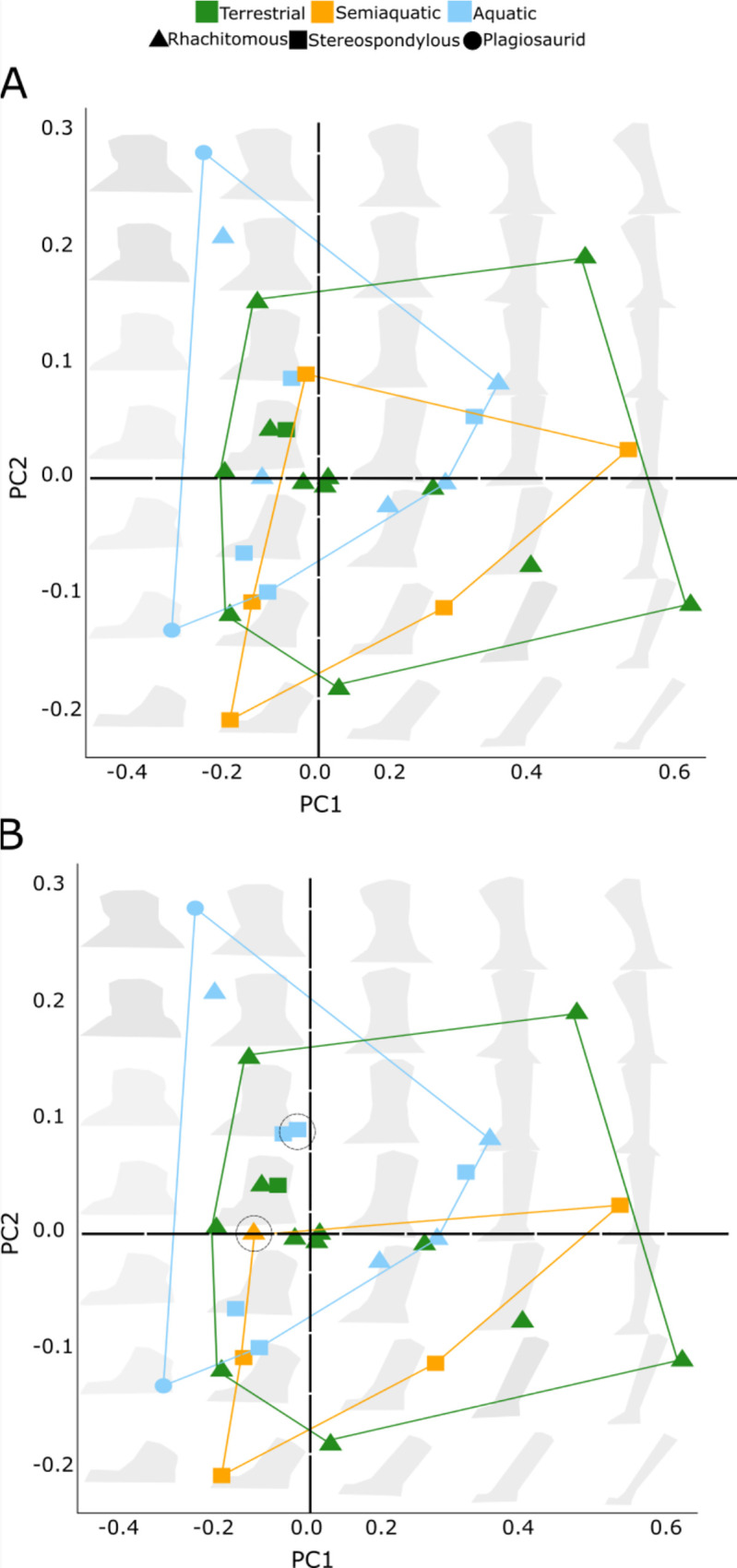
Morphospace for temnospondyl neural arches. Principle component analysis shows vertebral type distribution. **A**: “consensus” data set; **B**: “most recent” data set. Dashed circles represent changes between A and B. Convex hulls are grouped according to a priori habitat. Grey shapes are theoretical neural arches representing at a particular point in PC1 and PC2. Shape of the point represents the vertebral type.

We did not recover a significant relationship between neural arch form and environment preferences in temnospondyls (ANOVA P = 0.583, Phylogenetic ANOVA P = 0.876; Tables 2 and 3; Fig 2 and S4 Fig in S1 File). The first two principal components only comprise 42% and 20% the total variance respectively (S5 Table in S1 File). Additionally, there were no outliers of shape in our sample. Temnospondyls with positive PC1 scores feature axially elongated neural spines with the edge of the postzygapophysis located slightly more caudally than the distal extremity of the spine. Species with negative PC1 scores have axially shorter neural spines and postzygapophyses that are directed more caudally distal to the neural spine. There was no difference in our shape results between the “consensus” and the “most recent” data sets (Fig 2).

**Table 2:** Shape-ANOVA results.

	Neural Spine				Intercentra			
	Df	F	P	R^2^	Df	F	P	R^2^
Centroid Size	1	1.351	0.195	0.047	1	1.0006	0.368	0.032
Habitat	2	0.8155	0.583	0.059	2	7.3734	0.001*	0.337
Vertebral type	2	1.4725	0.148	0.102	2	2.9985	0.001*	0.171
Geologic Age	6	1.165	0.288	0.2412	5	1.7648	0.036*	0.246
Image Source	3	1.300	0.209	0.135	3	1.0273	0.404	0.099

Table for both neural arches and intercentra. Df, degrees of freedom; F, f-values; P, P-values, and R^2^ values.

**Table 3:** Phylogenetic-ANOVA results.

	Neural Spine				Intercentra			
	Df	F	P	R^2^	Df	F	P	R^2^
Centroid Size	1	0.5473	0.554	0.198	1	0.6341	0.656	0.0207
Habitat	2	0.4862	0.876	0.036	2	3.5434	0.002*	0.1963
Vertebral type	2	0.6945	0.626	0.051	2	2.0935	0.003*	0.1262
Geologic Age	6	0.4522	0.952	0.110	5	0.7722	0.758	0.1293
Image Source	3	1.395	0.205	0.144	3	1.4422	0.073	0.1528

Table for both neural arches and intercentra. Df, degrees of freedom; F, f-values; P, P-values, and R2 values

Phylogenetic generalized least squares analysis (PGLS) returned no significant relationships. Physignal produced a K value of 0.3877 and P = 0.397. ThreshBayes produced weak-to-nonexistent effect sizes and correlation coefficients for habitat and PC1, with r values of -0.078 for terrestrial temnospondyls, 0.11 for aquatic species, and -0.05 for semiaquatic taxa.

### Intercentrum shape

All our results support a strong relationship between habitat preference and intercentrum shape in temnospondyls. There was one outlier in our sample, *Nooxobeia gracilis*. This species falls outside the upper quartile for Procrustes distances from the mean shape. We conducted shape ANOVAs with and without *Nooxobeia gracilis* and there were no differences in significant relationships of the shape ANOVAs without *Nooxobeia gracilis* (S7 Table in S1 File). We discuss the results without *Nooxobeia gracilis* below.

**Fig 3:**
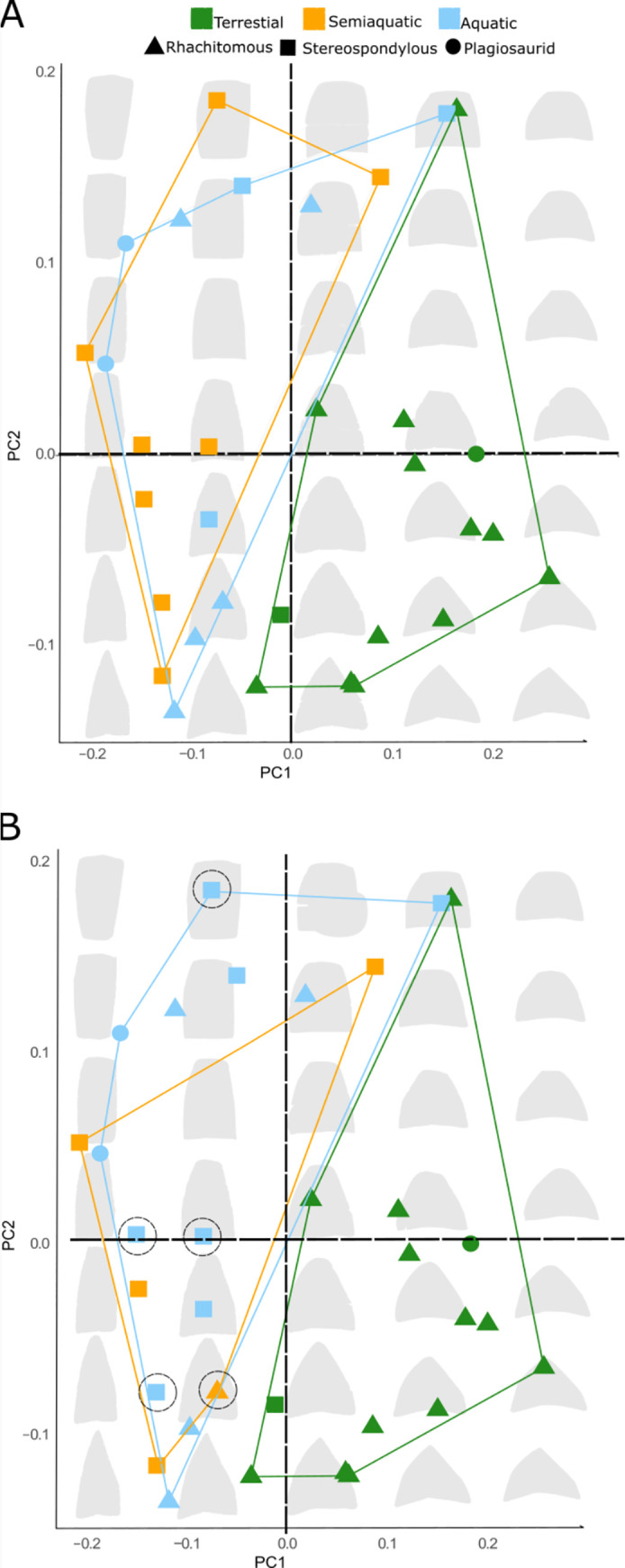
Morphospace for temnospondyl intercentra. Principle component analysis displaying vertebral type distribution. Convex hulls are grouped according to a priori habitat. **A**: “consensus” data set; **B**: “most recent” data set. Dashed circles represent changes between A and B. Grey shapes are theoretical intercentra representing at a particular point in PC1 and PC2. Shape of the point represents the vertebral type.

80% of the total variance was explained in the first two principal components (Fig 3; S6 Table in S1 File). Plots show distinct clusters in morphospace for terrestrial temnospondyls and a combined distribution of aquatic and semiaquatic temnospondyls, with almost complete separation along PC1 (Fig 3). ANOVAs on principal components showed statistically significant differences of PC scores of terrestrial taxa from aquatic and semiaquatic taxa on PC1, and between terrestrial and aquatic taxa on PC2. Both Phylogenetic ANOVAs and non-phylogenetic ANOVA of shape against habitat showed there is a statistically significant difference between intercentrum morphology among different habitats, and vertebral type (Tables 2 and 3, SI; p<0.001). The phylogenetic ANOVA returned significance between shape and geologic age (SI; p<0.005). Additionally, *Physignal* produced a significant (P = 0.001) phylogenetic signal K = 0.7168.

Principal component 1 describes intercentrum height, axial length, and ventral curvature. Species with intercentra characterized by reduced height and increased ventral curvature had positive PC1 scores and are previously inferred to be terrestrial (see methods, Fig 3). In contrast, aquatic and semiaquatic temnospondyls with negative scores on PC1 feature taller intercentra, consistent centra length, and flattening on the ventral surface.

The second PC describes intercentrum dorsal and ventral surface shape. Positive PC2 values characterize intercentra with dorsally flattened and craniocaudally elongated surfaces. Positive PC2 taxa also have a reduced ventral curvature, with the cranial and caudal extremal points more ventral than the rest of the centrum. Negative scores on PC2 indicate intercentra with the dorsal surface tapering to a point, resulting in a triangular shape. Terrestrial temnospondyls tend to have short intercentra with wide, curved bases and pointed dorsal surfaces. Aquatic and semi-aquatic species overlapped in shape, and both groups have tall intercentra with flat bases. There was no significant difference in our shape results between the consensus data set and the most recent data set (Fig 3).

There was a significant relationship between the general vertebral morphotypes and intercentra shapes (Tables 1 and 2). *ThreshBayes* revealed that habitat use has a moderate effect size for aquatic, and semiaquatic taxon morphology (r = -0.37 and r = -0.49 respectively). There was a very strong effect of size and high correlation between living on land and intercentrum shape (r = 0.92, r^2^ = 0.84). There are no significant differences in morphological disparity among terrestrial, semiaquatic, or aqautic morphologies.

### Habitat shifts in temnospondyls

**Fig 4:**
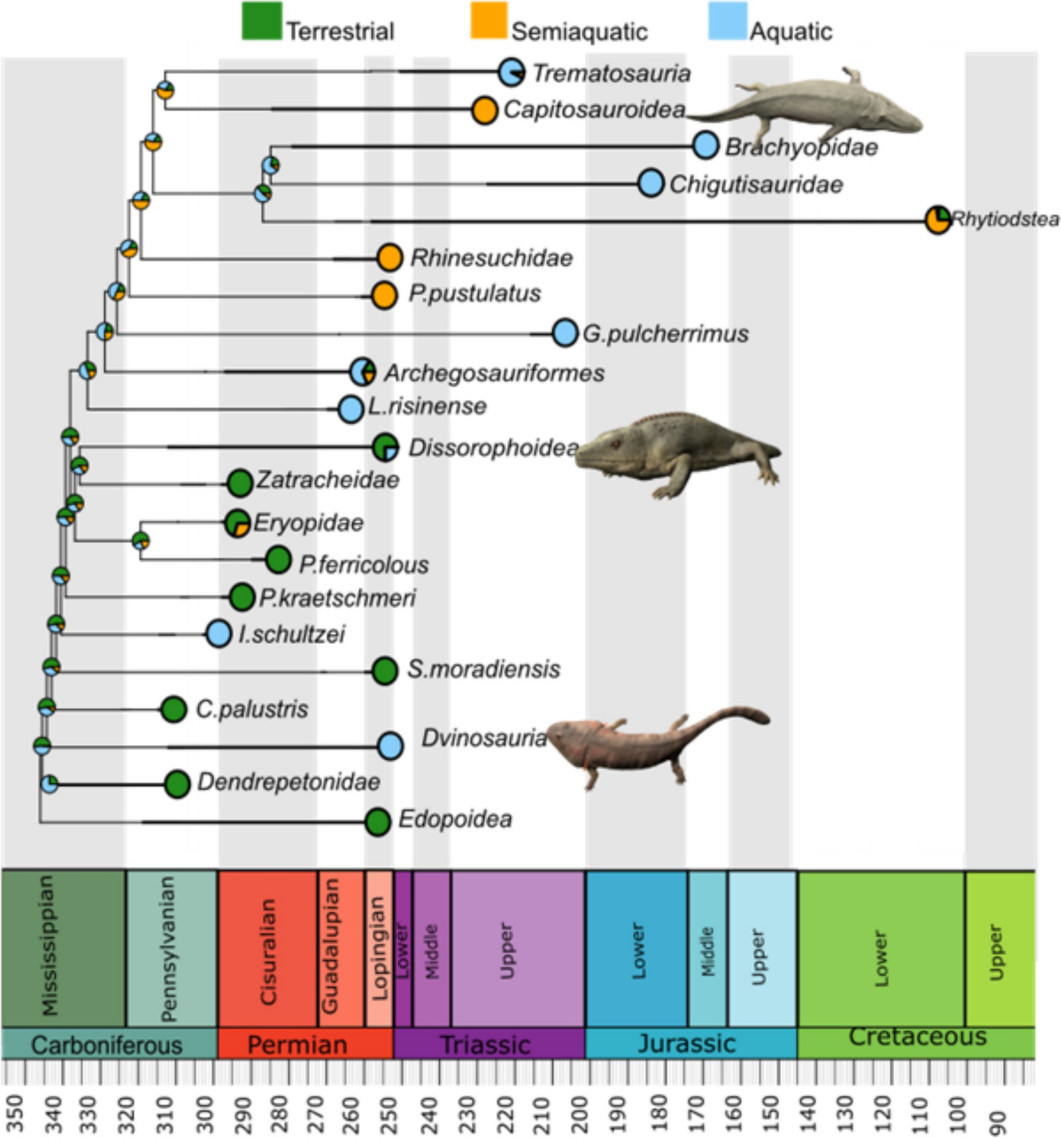
Phylogeny and habitat of temnospondyls. Tip colors are pooled prior probabilities for clades as gathered from literature and paleobiology database (PBDB; Supplementary Information). Node colors display posterior probabilities calculated from the best-fit (OU; terrestrial-semiaquatic-aquatic) mode in Ancthresh. Thick lines represent stratigraphic range. Reconstructions borrowed with permission from Nobu Tamura.

Across all tested sequences of initial habitat states, the Ornstein-Uhlenbeck (OU) model fit best with the lowest DIC values and a DIC weight of ~1 (S10 and S11 Tables in S1 File). The sequence terrestrial-semiaquatic-aquatic had the lowest DIC values under the OU model overall and is therefore the most probable of all the models. Regardless of the order of initial habitats used, ancestral nodes were always more likely to be terrestrial (Fig 4, S5 Fig in S1 File). In all cases, transitions into semiaquatic and aquatic habitats did occur frequently and independently among later lineages, with very little cost in terms of liability (Fig 4, S5 Fig in S1 File). Secondary terrestriality did occur in our dataset in the Lydekkerinidae and Laidleriidae.

## Discussion

Our shape data and ancestral state reconstruction demonstrate the high degree of temnospondyl vertebral diversity. The neural arches demonstrate no environmental, geologic, or size correlations. Conversely, intercentra correlate tightly with habitat and vertebral classification. Although intercentra shape are tightly associated with habitat preference, the environmental-morphological relationships are contrary to what was previously hypothesized (see below). We also demonstrate that the basal temnospondyls were terrestrial and reinvasions into aquatic environments required little evolutionary time or cost (as represented by low threshold liability values in our AncThresh analyses [68, 76]).

### Neural arch morphology is similar across taxa

Neural arches protect the spinal cord via the spinal canal. The neural arch also supports pre- and postzygapophyses and the neural spine. Previous studies hypothesized several morphological modifications to the latter two structures as reliable indicators of terrestriality, mainly by limiting trunk sag [16, 57, 87]. Our results counter previous assertions that neural arches are reliable indicators of terrestriality, including that spine morphologies correlated with increased rigidity are essential for locomotion on land [13, 16, 29, 88]. At least among temnospondyls, terrestrial and aquatic taxa have surprisingly similar neural arch morphologies–there is complete overlap between terrestrial and aquatic forms in most of our morphospace (Fig 2). Both terrestrial and aquatic taxa have ventrally-sloped prezygapophyses, although they are reduced in aquatic taxa (S7 Fig in S1 File). Few terrestrial temnospondyls, including those diverging near the base of the tree, have the flat spatulate facets suggested to resist trunk sag [21, 23].

It is possible that other neural arch traits and elements may have supported terrestriality, but are not captured by our landmarking scheme. For example, osteoderms in terrestrial dissorophids, might compensate for the lack of reinforcement [15, 21, 29, 89]. However, terrestrial taxa such as *Eryops megacephalus* and *Dendrerpeton acadianum* do not have distinct neural arch morphologies. The variation in neural arches in terrestrial taxa suggests that alternative effects of the neural arch in terrestriality should be investigated in the future.

Our results suggest that derived aquatic taxa such as *Mastodonosaurus giganteus* were likely descended from a semi-aquatic taxon (see ancestral state reconstruction below), and these taxa still retain terrestrial-like prezygapophyses. Variable prezygapophyseal articulations may reflect more nuanced aquatic life modes, and the requirements of these might be similar to that of terrestrial taxa. These similarities may be the cause of the substantial overlap in the neural arch morphospace. For example, *Peltobatrachus pustulatus* was described by Panchen in 1958 [90] as a terrestrial taxon partially due to its highly ossified vertebral column and flat spatulate prezygapophyses (among other terrestrial traits). However, after later investigations and additional discoveries, researchers determined that *P*. *pustulatus* was a bottom-walking taxon, and the supportive neural column was in response to its heavy dermal armor [91]. In our study we have demonstrated that other aquatic taxa have morphologies that increase intervertebral rigidity; thus, we reject previously held hypotheses about rigid spines being required only for terrestrial life. We could, therefore, imagine that benefits afforded by stiff intervertebral joints could also be used in aquatic taxa for various life modes. Heavily-reinforced intervertebral joints are already known in both living and extinct fishes of the same age as early temnospondyls (e.g., *Tarrasius problematicus*, [92]) as well as fully aquatic extant salamanders like *Amphiuma* [92, 93].

### Implications of neural arches for epaxial musculature

We were able to infer anatomical information about the epaxial muscles in temnospondyls based on neural spine morphology. Modern salamanders, functional homologs, and likely descendants of temnospondyls [24] have two main muscles that aid in lateral bending: the dorsalis trunci and the interspinalis [54]. The dorsalis trunci originates and inserts via transverse myosepta on the neural spines in modern taxa [8, 54, 57, 87]. However, the only myological reconstruction study conducted on temnospondyls [94] posits that the dorsalis trunci inserts on the transverse processes of the neural arch, a morphology not captured by this study. In his work, Olson [94] was surprised that all modern lissamphibians have a dorsalis trunci (longissimus dorsi) that inserts along the neural spine, and not the transverse processes. We propose that perhaps the dorsalis trunci did insert along the neural spine via myosepta (as seen in all modern lissamphibians), and such attachments would not preserve in the fossil record [94]. However, this requires further study. If the dorsalis trunci did indeed insert along the neural spine, as in modern amphibians, the effect of neural spine morphology and muscle force would be similar to the interspinalis muscle discussed below.

The interspinalis ("between spines") muscle, in both modern taxa and Olson’s [94] reconstruction, bridges the gap between adjacent neural spines. The muscle originates from the cranial end of one neural spine and inserts on the caudal surface of the cranially adjacent spine [94]. Muscle force can be amplified in two complementary ways: 1. by increasing the size of the muscle; and 2. by increasing its moment arm [95]. Below, we discuss first the increase in size of the muscle and then changes in moment arm.

In general, larger attachment areas usually indicate larger muscle mass and thus greater possible force [96]. For our study, we used centroid size as a marker for the area of the neural spine. In geometric morphometrics, centroid size is used as a measurement for an area almost universally because it is independent of scale, translation, or rotation. Interestingly, there was no discernable distinction between muscle attachment size and form between terrestrial and aquatic temnospondyls.

A moment arm is the perpendicular distance from the point of rotation to the line of force. For muscles of equivalent size, the larger the moment arm, the greater the output force that can be produced. If we assume that the force of the interspinalis muscle is the same and the neural spine rotates along its ventral surface, than its moment arms would be qualitatively larger for taxa with tall neural spines (positive PC1 scores) than with shorter neural spines (negative PC1 scores, and both PC2 scores). These differences among the principal components suggest high degrees of stabilizing forces from both the interspinalis and dorsalis trunci in taxa with high PC1 scores.

The cranial and caudal orientation of the spine also varies throughout the PC space. Traditional myology studies suggest that the more cranially angled a neural spine is, the more flexible is the intervertebral joint [45, 50]. However, this correlation is based on large dorsal interspinous ligaments found in mammals and, indeed, in taxa without this large ligament (i.e., crocodylians) the opposite morphological-stiffness relationship occurs (i.e., cranially oriented neural spines correlate with greater stiffness) [52]. Therefore, without additional morphological-stiffness studies on taxa outside of mammals, it is difficult to state how the neural spine angle affects local intervertebral joint stiffness in temnospondyls.

Our geometric morphometric study was conducted in 2D lateral view, and we found that terrestrial and aquatic temnospondyls overlap in their neural arch morphologies. However, by not incorporating the third dimension into our analyses, we may have missed some of the morphological complexity that could distinguish terrestrial and aquatic forms, or life modes. Some lateral bending is also controlled by muscles that insert along the ribs or transverse processes (subvertebralis pars lateralis and medialis, obliquus internus) [7, 55, 56]. However, because we did not characterize the transverse processes and ribs in this study, we cannot comment on these muscle groups. In the future, we suggest employing three-dimensional techniques whereby the neural arches and intercentra can be analyzed together.

### Intercentra morphology reflects environmental distribution and ecology

Intercentrum morphology is highly variable among temnospondyls, reflecting a range of potential intervertebral flexibility. However, our results show that distinct sets of intercentrum forms define terrestrial and aquatic species. Most terrestrial temnospondyls have short, axially-compressed or “spool-shaped” vertebrae with amphicoelous ends. In contrast, aquatic temnospondyls have disk-shaped intercentra that are taller than long. Biomechanical studies of aquatic and semi-aquatic tetrapods, including dolphins, ichthyosaurs, and extant crocodilians, have shown that discoid vertebrae have reduced axial flexibility relative to taxa with spool-shaped vertebrae [4, 52, 58]. Additionally, aquatic taxa range from amphicoelous vertebrae (low PC2 scores, e.g., *Trimerorhachis* and *Neldasaurus*) to opisthocoelous vertebrae (high PC2 scores, e.g., *Metoposaurus*) [16, 97]. Previous biomechanical studies found that opisthocoelous vertebrae increase stability between intervertebral joints [60, 98]. The disk-shaped intercentra with opisthocoelous ends were more rigid than the disk-shaped intercentra with amphicoelous ends. Although the intercentra shapes in our study are not perfect spools, as seen in many living fishes [99], we would still predict a more flexible vertebral column for terrestrial temnospondyls than the aquatic species in our study given their disk-like proportions [3, 49].

Our intercentrum results, including the amount of observed variability, are in stark contrast to many predictions that axial stiffness was a requirement of terrestrial locomotion [1, 10, 11, 13, 14, 29]. Some terrestrial taxa appear to have evolved additional morphological traits to increase axial rigidity, including fused osteoderms to stiffen the neural spine in dissorophids or decreasing vertebral counts from larval stage to adults in *Acanthostomatops vorax* [14, 15]. However, some large terrestrial forms (e.g., *Edops craigi*) exhibit no apparent accommodations, warranting further investigation into the effects and potential benefits of increased flexibility in terrestrial locomotion [76]. We note that the recent recreation of the fossil footprints of an early amniote, *Orobates pabsti*, required both axial flexibility and marked lateral movement of the spine [65]. In addition, modern salamanders use lateral axial undulations to move their limbs [7]. Thus, prior assumptions of extreme rigidity in the spines of early terrestrial tetrapods were likely erroneous.

Aquatic temnospondyls have less flexible intercentrum morphologies relative to terrestrial taxa. These stiffer morphologies are surprising as aquatic temnospondyls evolved from taxa with flexible morphologies. To increase axial flexibility, many aquatic temnospondyls increased the number of total vertebrae rather than modifed vertebral morphologies. For example, some aquatic temnospondyls (e.g., trimerorhachids, with 32 presacral vertebrae), may have evolved greater flexibility through high vertebral counts in their presacral series, providing more points of limited, but controlled bending in comparison to taxa with very low presacral counts (e.g., *Acanthostomatops vorax*, with 12 presacral vertebrae) [14, 71]. Conversely, other aquatic temnospondyls have decreased vertebral counts, implying greater rigidity and a different form of swimming (e.g., propulsion via pectoral limbs, carangiform tail-based swimming) [30], as previously suggested for some metoposaurs and archegosauriforms [30, 31].

### Pleurocentrum morphology, habitat, and locomotor implications

We were compelled to exclude pleurocentra from our morphometric analyses due to their incomplete appearance in the temnospondyl record. No empirical studies have investigated the role of pleurocentra on intervertebral motion in temnospondyls or terrestrial walking, and no modern tetrapods retain this feature. However, we can make some inferences about potential functions of pleurocentra on the basis of inferences in fossil fishes and the role of multipartite vertebrae in some modern fishes [11, 12, 100, 101]. Previous authors suggested that the presence of distinct pleurocentra in early sarcopterygians permitted greater torsional and bending flexibility and was required for lift in taxa with heterocercal tails such as *Osteolepis* [100]. Biomechanical studies of modern actinopterygians have supported this interpretation; e.g., the two mineralized centra per functional vertebral unit in Diplospondyly increased flexibility per vertebral joint [101–103]. Thus, temnospondyl taxa that retained distinct pleurocentra perhaps were more flexible than taxa with fused or absent pleurocentra. Later and larger rhizodont fishes independently evolved holospondylous vertebrae, i.e., with fused centra and central perforations for a continuous notochord, and previous authors suggest this change in vertebral type correlated to a greater axial stiffness required for the aquatic life mode of these taxa [100, 104]. Plagiosaurid and other later aquatic temnospondyls may have evolved fusion between the intercentra and pleurocentra for the same reason.

We can make some preliminary interpretations of the biomechanical forces underlying the distribution of pleurocentrum morphology in temnospondyls from different environments. Terrestrial temnospondyls with separate pleurocentra (e.g., *Eryops*, *Dendrepeton*) perhaps required some torsion and bending flexibility for their mode of walking. The presence or absence of pleurocentra suggests a difference in local vertebral joint stiffness between aquatic taxa with pleurocentra (e.g., *Trimerorhachis*, *Neldasaurus)* and taxa with minimal or complete loss of pleurocentra (e.g., *Mastodonosauru*, *Batrachosuchus)*. Similar to rhizodont fishes, later aquatic taxa required greater stabilization against torsion and bending motions. It is worth noting that *Doleserpeton* had a flexible intercentrum morphology but lacks pleurocentra [105]. This taxon may have exhibited a biomechanical compromise, increasing the stability of an intervertebral joint while maintaining some level of flexibility. We suggest future empirical studies investigating pleurocentra effects on intervertebral range of motion. Although we do not yet know the role of pleurocentra or multipartite vertebrae on temnospondyl motion, our results demonstrate that taxa frequently converge on intercentrum shapes expressive of their environment and life mode.

Regardless of the role of pleurocentra, our results suggest a strong link between intercentrum shape alone and ecological differentiation within groups sharing aquatic life modes. Semiaquatic forms cluster near the origin of PC2, whereas terrestrial and aquatic forms show a wider distribution (Fig 3). Fully aquatic temnospondyls with extreme positive scores, such as *Plagiosuchus*, have been previously designated as benthic "bottom-walkers" based on other characteristics such as pachyostotic ribs and heavily ossified limbs [106, 107]. In contrast, taxa such as *Trimerorhachis* and *Neldasaurus*, which have been reported as mid-water swimmers, have extremely negative PC2 scores (Fig 3) [30, 31]. It is important to note that *Trimerorhachis* and *Neldasaurus* are secondarily aquatic taxa derived from a terrestrial ancestor (see ancestral state reconstruction and [71]). Despite their ancestry, both taxa still converge on aquatic intercentra forms.

**Fig 5:**
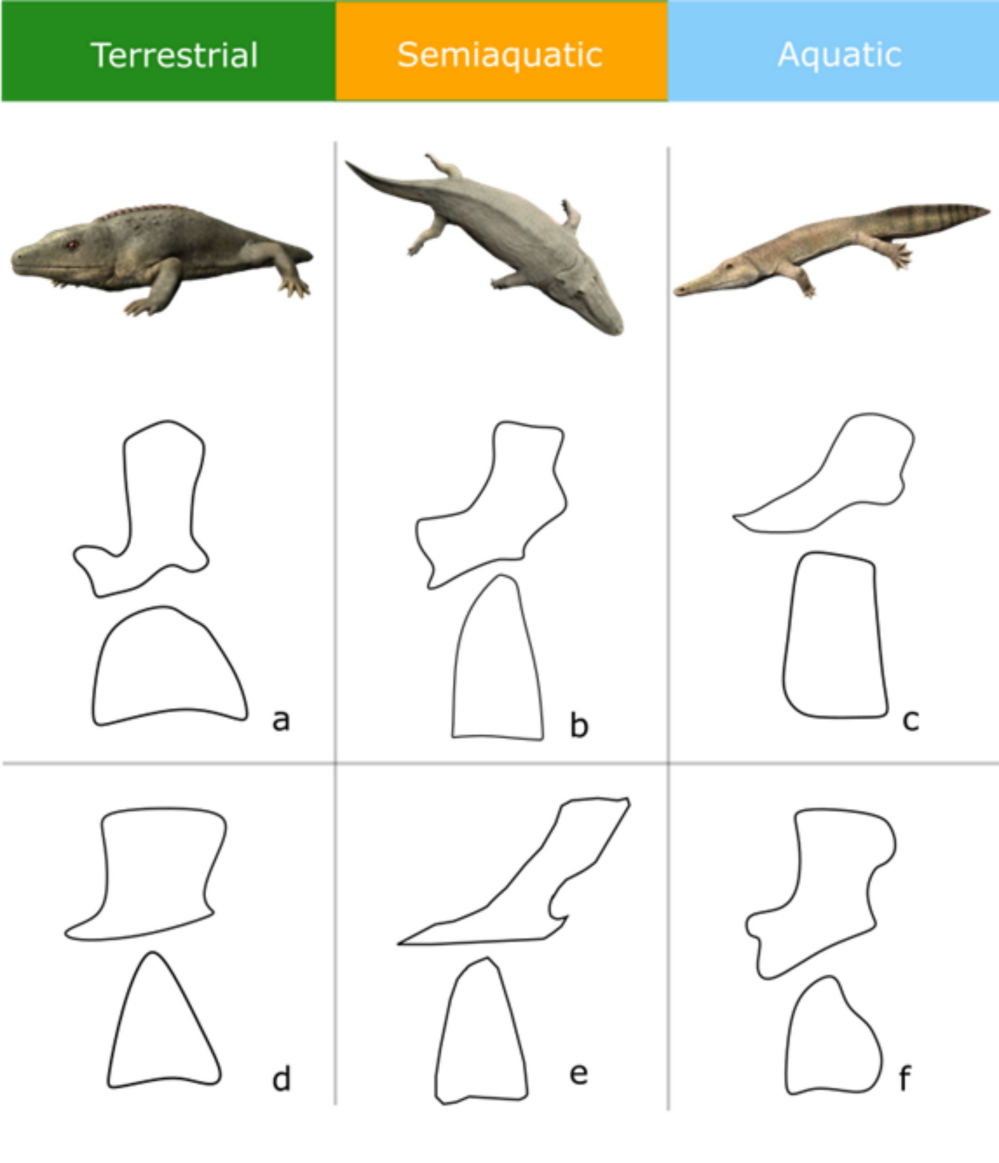
Traces of neural arch and intercentra shape convergence characterized by environment. *Top row*: *Reconstructions borrowed with permission from Nobu Tamura*. *From left to right*: *Cacops aspidephorus Paracyclotosaurus davidi*, *Archegosaurus decheni*. *Middle row*: *a) Cacops aspidephorus* (ancestrally terrestrial); b) *Paracyclotosaurus davidi* (ancestrally semiaquatic); c) *Metoposaurus diagnosticus* (ancestrally aquatic). Bottom row: d) *Lydekkerina huxleyi* (secondarily terrestrial); e) *Mastodonsaurus giganteus* (variation on semiaquatic form, note no secondarily semiaquatic taxa were reported from this study); f) *Archegosaurus decheni* (secondarily aquatic).

Within terrestrial taxa, some secondarily terrestrial temnospondyls forms retain “aquatic” features such as tabular horns and palatine vacuities from a recent common aquatic ancestor. *Laidleria gracilis* and *Lydekkerina huxleyi* are both secondarily terrestrial stereospondyls which exhibit derived flexible terrestrial vertebrae from the plesiomorphic aquatic and stiff condition (Fig 3). They both secondarily evolved spool-like intercentra, suggesting convergence on a vertebral form adapted for terrestrial locomotion. Repeated convergence on common intercentrum forms in aquatic and terrestrial taxa suggests selective pressure for distinct morphologies in separate environments, likely the result of specific functional and ecological requirements (Fig 5).

### Temnospondyls were likely ancestrally terrestrial

Early temnospondyls were likely terrestrial as shown by our ancestral state reconstructions. The mega-tree in our study [66] and the most recent phylogeny of Temnospondyli [32] both generated arrangements with edopids (*Edops*, *Cochleosaurus*, *Chenoprosopus*, *Nigerpeton*) as the sister group to other temnospondyls. *Edops*, *Cochleosaurus*, and *Chenoprosopus* have reduced lateral lines, and finite element analyses suggest they were terrestrial feeders [26, 108, 109]. The intercentrum of *Edops* clustered with other terrestrial species in our morphospace. In contrast, *Nigerpeton* has an enclosed lateral line, an autapomorphy for this genus, suggesting an aquatic lifestyle [109]. However, this aquatic genus occurs late in the edopid record (late Permian), and so did not change the probability distribution favoring a terrestrial ancestor (65% terrestrial, 2% aquatic) in edopids and therefore the probability of a terrestrial ancestor in Temnospondyli. Our study confirms previous hypotheses that temnospondyls were ancestrally terrestrial and the Dvinosauria were early, secondarily aquatic temnospondyls [71]. Additionally, we demonstrate eryopids likely had a terrestrial ancestor, challenging previous studies [110]. As per the most recent phylogeny for early tetrapods of Pardo et al. (2017), it appears that there was one transition from aquatic to terrestrial environments for tetrapods as a whole [24]. Early terrestrial temnospondyls would have had the forms previously discussed as axially flexible.

## Conclusions

Our contribution presents a novel approach to viewing vertebral morphology in early tetrapods and testing hypotheses about the form and function in fossil groups. This study quantified neural arch and intercentrum shape in temnospondyls, or in any group of Paleozoic tetrapods, more comprehensively than previous works. It is also the first to use a geometric morphometrics and phylogenetic comparative methods to both fossil vertebral form and its relationship with habitat transitions in early tetrapods. We have demonstrated that functionally terrestrial temnospondyls exhibited more flexibility than previously described, whereas aquatic taxa were characterized by intercentra classically considered to be rigid. Some aquatic taxa may have increased the vertebral number to regain flexibility required for swimming. Intercentra forms were tightly correlated with both habitat and classic vertebral type. However, taxa with similar vertebral types, like the aquatic rhachitomous *Trimerorhachis* and the terrestrial rhachitomous *Edops*, clustered within their environments rather than with one another (Fig 3, S6 Fig in S1 File). This distribution suggests that the vertebral types posited by Romer and used through early tetrapod literature are not useful for describing the structure and arrangement of vertebrae.

In sum, our results suggest that intercentrum form is a more reliable, objective indicator of habitat use and locomotion in early tetrapods than other, widely-used traits like neural spines and zygapophyses. While we did not include pleurocentra in our analysis, it is possible that these results can be generalized to other groups in which other vertebral elements serve a similar role to the intercentrum. We therefore recommend in-depth investigations on both pleurocentra and intercentra as combined units, and a much-needed conversation as to functional homology in multipartite vertebrae.

## Supporting information

S1 File (PDF)

S1 Data (ZIP)

## References

[1] Clack JA. Gaining ground: the origin and evolution of tetrapods. Indiana University Press; 2012. 2^nd^ ed.

[2] Pierce SE, Hutchinson JR, Clack JA. Historical Perspectives on the Evolution of Tetrapodomorph Movement. Integrative and Comparative Biology. 2013;53: 209–23. doi: 10.1093/icb/ict022 23624864

[3] Buchholtz EA. Vertebral osteology and swimming style in living and fossil whales (Order: Cetacea). Journal of Zoology. 2001;253: 175–190.

[4] Motani R, You H, McGowan C. Eel-like swimming in the earliest ichthyosaurs. Nature. 1996 Jul;382(6589):347–8.

[5] Panchen AL. The axial skeleton of the labyrinthodont *Eogyrinus attheyi*. Journal of Zoology. 1966;150:199–222.

[6] Moritz S, Schilling N. Fiber‐type composition in the perivertebral musculature of lizards: Implications for the evolution of the diapsid trunk muscles. Journal of morphology. 2013;274:294–306. doi: 10.1002/jmor.20091 23115131

[7] Karakasiliotis K, Schilling N, Cabelguen JM, Ijspeert AJ. Where are we in understanding salamander locomotion: biological and robotic perspectives on kinematics. Biological cybernetics. 2013;107(5):529–44. doi: 10.1007/s00422-012-0540-4 23250621

[8] Deban SM, Schilling N. Activity of trunk muscles during aquatic and terrestrial locomotion in *Ambystoma maculatum*. Journal of Experimental Biology. 2009;212:2949–59. doi: 10.1242/jeb.032961 19717677

[9] Pierce SE, Ahlberg PE, Hutchinson JR, Molnar JL, Sanchez S, Tafforeau P, et al. Vertebral architecture in the earliest stem tetrapods. Nature. 2013;494:226–9. doi: 10.1038/nature11825 23334417

[10] DeFauw SL. Temnospondyl amphibians: a new perspective on the last phases in the evolution of the Labyrinthodontia. Michigan Academician. 1989;21: 7–32.

[11] Panchen AL. The Origin and Early Evolution of Tetrapod Vertebrae. Linnean Society Symposium: Problems in vertebrate evolution. 1977; 289–318.

[12] Parrington FR. The vertebrae of early tetrapods. Colloques International du Centre National de la Researche Scientifique. 1967;163: 271–272.

[13] Rockwell H, Evans GF, Pheasant HC. The comparative morphology of the vertebrate spinal column. Its form as related to function. Journal of Morphology. 1938;63: 87–117.

[14] Witzmann F, Schoch RR. Skeletal development of the temnospondyl *Acanthostomatops vorax* from the Lower Permian Döhlen Basin of Saxony. Earth and Environmental Science Transactions of the Royal Society of Edinburgh. 2005;96:365–85.

[15] Dilkes DW. Comparison and biomechanical interpretations of the vertebrae and osteoderms of *Cacops aspidephorus* and *Dissorophus multicinctus* (Temnospondyli, Dissorophidae). Journal of Vertebrate Paleontology. 2009;29: 1013–21.

[16] Warren A, Snell N. The postcranial skeleton of Mesozoic temnospondyl amphibians: a review. Alcheringa. 1991;15:43–64.

[17] Danto M, Witzmann F, Fröbisch NB. Vertebral development in Paleozoic and Mesozoic tetrapods revealed by paleohistological data. PloS One. 2016;11:e0152586. doi: 10.1371/journal.pone.0152586 27074015 PMC4830443

[18] Konietzko‐Meier D, Danto M, Gądek K. The microstructural variability of the intercentra among temnospondyl amphibians. Biological Journal of the Linnean Society. 2014;112: 747–764.

[19] Danto M, Witzmann F, Pierce SE, Fröbisch NB. Intercentrum versus pleurocentrum growth in early tetrapods: A paleohistological approach. Journal of Morphology. 2017;27:1262–83 doi: 10.1002/jmor.20709 28517044

[20] Holmes R. Functional interpretations of the vertebral structure in Paleozoic labyrinthodont amphibians. Historical Biology. 1989;2: 111–24.

[21] Dilkes D, Brown LE. Biomechanics of the vertebrae and associated osteoderms of the Early Permian amphibian *Cacops aspidephorus*. Journal of Zoology. 2007;271: 396–407.

[22] Dilkes D. A new trematopsid amphibian (Temnospondyli: Dissorophoidea) from the Lower Permian of Texas. Paleontology. 1990;10: 222–243.

[23] Olson EC. The exploitation of land by early tetrapods. Morphology and biology of reptiles. 1976; 1–30.

[24] Pardo JD, Szostakiwskyj M, Ahlberg PE, Anderson JS. Hidden morphological diversity among early tetrapods. Nature. 2017;546:642–5. doi: 10.1038/nature22966 28636600

[25] Ruta M, Benton MJ. Calibrated diversity, tree topology and the mother of mass extinctions: the lesson of temnospondyls. Palaeontology. 2008;51:1261–88.

[26] Fortuny J, Marcé‐Nogué J, De Esteban-Trivigno S, Gil L, Galobart À. Temnospondyli bite club: ecomorphological patterns of the most diverse group of early tetrapods. Journal of Evolutionary Biology. 2011;24:2040–54. doi: 10.1111/j.1420-9101.2011.02338.x 21707813

[27] Fröbisch NB, Olori JC, Schoch RR, Witzmann F. Amphibian development in the fossil record. In Seminars in Cell & Developmental Biology 2010;21:424–431. Academic Press. 19913630 10.1016/j.semcdb.2009.11.001

[28] Schoch RR. Life cycles, plasticity and palaeoecology in temnospondyl amphibians. Palaeontology. 2014;57:517–29.

[29] Reisz R, Schoch R, Anderson J. The armoured dissorophid *Cacops* from the Early Permian of Oklahoma and the exploitation of the terrestrial realm by amphibians. Naturwissenschaften. 2009;96: 789–96. doi: 10.1007/s00114-009-0533-x 19347261

[30] Sulej TO. Osteology, variability, and evolution of *Metoposaurus*, a temnospondyl from the Late Triassic of Poland. Polnica. 2007;64: 29–139.

[31] Witzmann F, Brainerd E. Modeling the physiology of the aquatic temnospondyl *Archegosaurus decheni* from the early Permian of Germany. Fossil Record. 2017;20: 105–127.

[32] Schoch RR. The evolution of major temnospondyl clades: an inclusive phylogenetic analysis. Journal of Systematic Palaeontology. 2013;11:673–705.

[33] Parrington FR. Intercentra: a possible functional interpretation. Linnean Society Symposium Series. 1977;4.

[34] Gregory WK. The upright posture of man: A review of its origin and evolution. Proceedings of the American Philosophical Society. 1928;67: 339–77.

[35] Gadow H. On the Evolution of the Vertebral Column of Amphibia and Amniota. Proceedings of the Royal Society of London. 1895;187: 257–259.

[36] Willia EE. Gadow’s arcualia and the development of tetrapod vertebrae. Quarterly Review of Biology. 1959;34: 1–32. 10.1086/402573 13658354

[37] Cope ED. On the intercentrum of the terrestrial Vertebrata. Transactions of the American Philosophical Society. 1888;16:243–53.

[38] Säve-Söderbergh G. Some points of view concerning the evolution of the vertebrates and the classification of this group. Arkiv för zoologi. 1934;26: 1–20.

[39] Romer AS. Osteology of the Reptiles. Chicago, Illinois: University of Chicago; 1956.

[40] Romer AS. Review of the Labyrinthodontia. Comparative Zoology. 1947:1–37.

[41] Shishkin M.A. Evolution of early amphibians (Plagiosauroidea). Paleontologiceskogo Instituta Akademiya Nauk USSR. 1987;225: 1–143.

[42] Panchen AL. The homologies of the Labyrinthodont centrum. Evolution. 1967;21: 24. doi: 10.1111/j.1558-5646.1967.tb00127.x 28556114

[43] Warren A. Laidleria uncovered: a redescription of *Laidleria gracilis* Kitching (1957), a temnospondyl from the Cynognathus Zone of South Africa. Zoological Journal of the Linnean Society. 1998;122(1–2):167–85.

[44] Goodrich ES. Studies on the structure & development of vertebrates. 1986.

[45] Boszczyk BM, Boszczyk AA, Putz R. Comparative and functional anatomy of the mammalian lumbar spine. The Anatomical Record. 2001;264: 157–68. doi: 10.1002/ar.1156 11590593

[46] Buchholtz EA, Wolkovich EM, Cleary RJ. Vertebral osteology and complexity in Lagenorhynchus acutus (Delphinidae) with comparison to other delphinoid genera. Marine Mammal Science. 2005;21:411–28.

[47] Pierce SE, Clack JA, Hutchinson JR. Comparative axial morphology in pinnipeds and its correlation with aquatic locomotory behaviour. Journal of Anatomy. 2011;219:502–14. doi: 10.1111/j.1469-7580.2011.01406.x 21668895 PMC3196755

[48] Russo GA. Prezygapophyseal articular facet shape in the catarrhine thoracolumbar vertebral column. American journal of physical anthropology. 2010;142:600–12. doi: 10.1002/ajpa.21283 20310062

[49] Buchholtz EA, Schur SA. Vertebral osteology in Delphinidae (Cetacea). Zoological Journal of the Linnean Society. 2004;140:383–401.

[50] Slijper E. Comparative biologic anatomical investigations on the vertebral column and spinal musculature of mammals. Tweede sectie. 1946;17(5):1–28.

[51] Shapiro LJ. Morphological and functional differentiation in the lumbar spine of lorisids and galagids. American Journal of Primatology: Official Journal of the American Society of Primatologists. 2007;69(1):86–102. doi: 10.1002/ajp.20329 17171674

[52] Molnar JL, Pierce SE, Hutchinson JR. An experimental and morphometric test of the relationship between vertebral morphology and joint stiffness in Nile crocodiles (*Crocodylus niloticus*). Journal of Experimental Biology. 2014;217: 758–68. 10.1242/jeb.089904 24574389

[53] Carrizo L V, Tulli MJ, Santos DA, Abdala V. Interplay between postcranial morphology and locomotor types in Neotropical sigmodontine rodents. Journal of Anatomy. 2014;224: 469–481. doi: 10.1111/joa.12152 24372154 PMC4098680

[54] O’Reilly JC, Summers AP, Ritter DA. The evolution of the functional role of trunk muscles during locomotion in adult amphibians. American Zoologist. 2000; 40:123–35.

[55] Simons RS, Brainerd EL. Morphological variation of hypaxial musculature in salamanders (Lissamphibia: Caudata). Journal of morphology. 1999;241:153–64. doi: 10.1002/(SICI)1097-4687(199908)241:2&lt;153::AID-JMOR5&gt;3.0.CO;2-E 10420161

[56] Brainerd EL, Azizi E. Muscle fiber angle, segment bulging and architectural gear ratio in segmented musculature. The Journal of Experimental Biology. 2005;208: 3249–61. doi: 10.1242/jeb.01770 16109887

[57] Schilling N. Evolution of the axial system in craniates: morphology and function of the perivertebral musculature. Frontiers in Zoology. 2011;8: 1–19. doi: 10.1186/1742-9994-8-1 21306656 PMC3041741

[58] Viglino M, Flores DA, Ercoli MD, Álvarez A. Patterns of morphological variation of the vertebral column in dolphins. J Zool. 2014;294: 267–277.

[59] Long JH, Pabst DA, Shepherd WR, McLellan WA. Locomotor design of dolphin vertebral columns: bending mechanics and morphology of *Delphinus delphis*. The Journal of Experimental Biology. 1997;200: 65–81. 9023994 10.1242/jeb.200.1.65

[60] Fronimos JA, Wilson JA. Concavo-convex intercentral joints stabilize the vertebral column in sauropod dinosaurs and crocodylians. Ameghiniana. 2017;54:151–76.

[61] Nowroozi BN, Harper CJ, Kegel DB, Adriaens D, Brainerd EL. Regional variation in morphology of vertebral centra and intervertebral joints in striped bass, *Morone saxatilis*. Journal of Morphology. 2012;273: 441–52. doi: 10.1002/jmor.11034 22109664

[62] Van Damme R, Vanhooydonck B. Speed versus manoeuvrability: association between vertebral number and habitat structure in lacertid lizards. Journal of Zoology. 2002;258(3):327–34.

[63] Long JH, Krenitsky NM, Roberts SF, Hirokawa J, De Leeuw J, Porter ME. Testing biomimetic structures in bioinspired robots: How vertebrae control the stiffness of the body and the behavior of fish-like swimmers. Integrated and Comparative Biology. 2011;51: 158–175. 10.1093/icb/icr020 21576117

[64] Shapiro LJ, Simons CVM. Functional aspects of strepsirrhine lumbar vertebral bodies and spinous processes. Journal of human evolution. 2002;42: 753–83. doi: 10.1006/jhev.2002.0560 12069508

[65] Nyakatura JA, Melo K, Horvat T, Karakasiliotis K, Allen VR, Andikfar A, et al. Reverse-engineering the locomotion of a stem amniote. Nature. 2019 Jan;565(7739):351–5. doi: 10.1038/s41586-018-0851-2 30651613

[66] Ruta M, Pisani D, Lloyd GT, Benton MJ. A supertree of Temnospondyli: cladogenetic patterns in the most species-rich group of early tetrapods. Proceedings of the Royal Society B: Biological Sciences. 2007;274:3087–95. doi: 10.1098/rspb.2007.1250 17925278 PMC2293949

[67] Bell MA, Lloyd GT. strap: an R package for plotting phylogenies against stratigraphy and assessing their stratigraphic congruence. Palaeontology. 2015;58:379–89.

[68] Sallan L, Friedman M, Sansom RS, Bird CM, Sansom IJ. The nearshore cradle of early vertebrate diversification. Science. 2018 Oct 26;362(6413):460–4. doi: 10.1126/science.aar3689 30361374

[69] Kuiper RM, Buskens V, Raub W, Hoijtink H. Combining statistical evidence from several studies: A method using Bayesian updating and an example from research on trust problems in social and economic exchange. Sociological Methods & Research. 2013;42:60–81.

[70] Sutton AJ, Abrams KR. Bayesian methods in meta-analysis and evidence synthesis. Statistical methods in medical research. 2001;10:277–303. doi: 10.1177/096228020101000404 11491414

[71] Pawley KA. The postcranial skeleton of *Trimerorhachis insignis* Cope, 1878 (Temnospondyli: Trimerorhachidae): a plesiomorphic temnospondyl from the Lower Permian of North America. Journal of Paleontology. 2007 Sep 1;81(5):873–94.

[72] Witzmann F. Developmental patterns and ossification sequence in the Permo-Carboniferous temnospondyl Archegosaurus decheni (Saar-Nahe Basin, Germany). Journal of Vertebrate Paleontology. 2006 Mar 30;26(1):7–17.

[73] Schoch RR. Early larval ontogeny of the Permo-Carboniferous temnospondyl *Sclerocephalus*. Palaeontology. 2003;46: 1055–1072.

[74] Adams DC, Otárola‐Castillo E. geomorph: an R package for the collection and analysis of geometric morphometric shape data. Methods in Ecology and Evolution. 2013;4:393–9.

[75] Revell LJ. phytools: an R package for phylogenetic comparative biology (and other things). Methods in ecology and evolution. 2012;3:217–23.

[76] Revell LJ. Ancestral character estimation under the threshold model from quantitative genetics. Evolution. 2014;68:743–59. doi: 10.1111/evo.12300 24152239

[77] Felsenstein J. Using the quantitative genetic threshold model for inferences between and within species. Philosophical Transactions of the Royal Society of London B: Biological Sciences. 2005;360: 1427–34. doi: 10.1098/rstb.2005.1669 16048785 PMC1569509

[78] Falconer DS. The inheritance of liability to certain diseases, estimated from the incidence among relatives. Annals of human genetics. 1965;29:51–76.

[79] Fruciano C. Measurement error in geometric morphometrics. Development genes and evolution. 2016 Jun 1;226(3):139–58. doi: 10.1007/s00427-016-0537-4 27038025

[80] Cardini A. Missing the third dimension in geometric morphometrics: How to assess if 2D images really are a good proxy for 3D structures? Hystrix. 2014;25: 73–81.

[81] Pennell MW, Eastman JM, Slater GJ, Brown JW, Uyeda JC, Fitzjohn RG, et al. geiger v2. 0: an expanded suite of methods for fitting macroevolutionary models to phylogenetic trees. Bioinformatics. 2014;30:2216–8. doi: 10.1093/bioinformatics/btu181 24728855

[82] Watanabe A. How many landmarks are enough to characterize shape and size variation? PloS one. 2018;13(6):e0198341. doi: 10.1371/journal.pone.0198341 29864151 PMC5986137

[83] Olsen AM. Feeding ecology is the primary driver of beak shape diversification in waterfowl. Functional Ecology. 2017;31:1985–95.

[84] Jolliffe IT. Discarding variables in a principal component analysis. I: Artificial data. Journal of the Royal Statistical Society: Series C (Applied Statistics). 1972;21:160–73.

[85] Randau M, Goswami A, Hutchinson JR, Cuff AR, Pierce SE. Cryptic complexity in felid vertebral evolution: shape differentiation and allometry of the axial skeleton. Zoological Journal of the Linnean Society. 2016;178: 183–202.

[86] Schilling N, Deban SM. Fiber‐type distribution of the perivertebral musculature in *Ambystoma*. Journal of Morphology. 2010;271(2):200–14. doi: 10.1002/jmor.10791 19708065

[87] Bennett WO, Simons RS, Brainerd EL. Twisting and bending: the functional role of salamander lateral hypaxial musculature during locomotion. Journal of Experimental Biology. 2001 Jun 1;204(11):1979–89. 11441039 10.1242/jeb.204.11.1979

[88] Gál JM. Mammalian spinal biomechanics. II. Intervertebral lesion experiments and mechanisms of bending resistance. The Journal of Experimental Biology. 1993;174: 281–297. 8440969 10.1242/jeb.174.1.281

[89] Schoch RR. Character distribution and phylogeny of the dissorophid temnospondyls. Fossil Record. 2012;15:121–37.

[90] Panchen AL. A new armoured amphibian from the Upper Permian of East Africa. Philosophical Transactions of the Royal Society of London. Series B, Biological Sciences. 1959;242:207–81.

[91] Witzmann F, Soler‐Gijón R. The bone histology of osteoderms in temnospondyl amphibians and in the chroniosuchian Bystrowiella. Acta Zoologica. 2010 Jan;91(1):96–114.

[92] Sallan LC. Tetrapod-like axial regionalization in an early ray-finned fish. Proceedings of the Royal Society B: Biological Sciences. 2012 Aug 22;279(1741):3264–71. doi: 10.1098/rspb.2012.0784 22628471 PMC3385743

[93] Gardner JD. The fossil salamander Proamphiuma cretacea Estes (Caudata; Amphiumidae) and relationships within the Amphiumidae. Journal of Vertebrate Paleontology. 2003;23: 769–782.

[94] Olson E. The dorsal axial musculature of certain primitive permian tetrapods. Journal of Morphology. 1936;59: 265–311.

[95] Biewener AA. Scaling body support in mammals: limb posture and muscle mechanics. Science. 1989 Jul 7;245(4913):45–8. doi: 10.1126/science.2740914 2740914

[96] Molnar JL, Pierce SE, Bhullar BA, Turner AH, Hutchinson JR. Morphological and functional changes in the vertebral column with increasing aquatic adaptation in crocodylomorphs. Royal Society Open Science. 2015 Nov 1;2(11):150439. doi: 10.1098/rsos.150439 26716001 PMC4680616

[97] Witzmann F, Rothschild BM, Hampe O, Sobral G, Gubin YM, Asbach P. Congenital malformations of the vertebral column in ancient amphibians. Anatomia, histologia, embryologia. 2014 Apr;43(2):90–102. doi: 10.1111/ahe.12050 23551141

[98] Salisbury SW, Frey E. Crocodilian biology and evolution. Surrey Beatty & Sons; 2000.

[99] Peskin B, Henke K, Cumplido N, Treaster S, Harris MP, Bagnat M, et al. Notochordal signals establish phylogenetic Identity of the teleost spine. Current Biology. 2020 Jul 20;30(14):2805–14. doi: 10.1016/j.cub.2020.05.037 32559448 PMC8159021

[100] Laerm J. On the origin of rhipidistian vertebrae. Journal of Paleontology. 1979 Jan 1:175–86.

[101] Maxwell EE, Furrer H, Sánchez-Villagra MR. Exceptional fossil preservation demonstrates a new mode of axial skeleton elongation in early ray-finned fishes. Nature Communications. 2013 Oct 7;4(1):1–7. doi: 10.1038/ncomms3570 24096879

[102] Arratia G, Schultze HP, Casciotta J. Vertebral column and associated elements in dipnoans and comparison with other fishes: development and homology. Journal of Morphology. 2001 Nov;250(2):101–72. doi: 10.1002/jmor.1062 11746457

[103] Schaeffer B. Osteichthyan vertebrae. Zoological journal of the Linnean Society. 1967 Oct 1;47(311):185–95.

[104] Andrews SM, Westoll TS. XII.—The Postcranial Skeleton of Rhipidistian Fishes Excluding Eusthenopteron. Transactions of the Royal Society of Edinburgh. Royal Society of Edinburgh Scotland Foundation; 1970;68:391–489.

[105] Sigurdsen T, Bolt JR. The Lower Permian amphibamid Doleserpeton (Temnospondyli: Dissorophoidea), the interrelationships of amphibamids, and the origin of modern amphibians. Journal of Vertebrate Paleontology. 2010 1;30(5):1360–77.

[106] Konietzko-Meier D, Schmitt A. A histological study of a femur of *Plagiosuchus*, a Middle Triassic temnospondyl amphibian from southern Germany, using thin sections and micro-CT scanning∙. Netherlands Journal of Geosciences. 2013;92:97–108.

[107] Konietzko-Meier D, Bodzioch A, Sander PM. Histological characteristics of the vertebral intercentra of *Metoposaurus diagnosticus* (Temnospondyli) from the Upper Triassic of Krasiejów (Upper Silesia, Poland). Earth and Environmental Science Transactions of the Royal Society of Edinburgh. 2013;103(3–4):237–50.

[108] Romer AS, Witter R. *Edops*, a primitive rhachitomous amphibian from the Texas red beds. The Journal of Geology. 1942;50: 873–89.

[109] Steyer JS, Damiani R, Sidor CA, O’Keefe FR, Larsson HC, Maga A, et al. The vertebrate fauna of the Upper Permian of Niger. IV. *Nigerpeton ricqlesi* (Temnospondyli: Cochleosauridae), and the edopoid colonization of Gondwana. Journal of Vertebrate Paleontology. 2006;26:18–28.

[110] Werneburg R, Berman DS. Revision of the Aquatic Eryopid Temnospondyl Glaukerpeton avinoffi Romer, 1952, from the Upper Pennsylvanian of North America. Annals of Carnegie Museum. 2012;81:33–60.

