# Supplementary material for: Early amphibians evolved distinct vertebrae for habitat invasions
Source: PLoS One. 2021 Jun 9;16(6):e0251983. doi: 10.1371/journal.pone.0251983 (PMC8189462; doi:10.1371/journal.pone.0251983)
Supplement: S1 File — (PDF) [file pone.0251983.s001.pdf]

S1 File

Supplemental Information for

Early Tetrapods Repeatedly Evolved Distinct Vertebrae for Life on Land

Aja M. Carter [1a], S. Tonia Hsieh[2], Peter Dodson[1,3], And Lauren Sallan[1]

Aja M. Carter
Email: caja@seas.upenn.edu

Figs. S1 to S6
Tables S1 to S12

All results are summarized in the Supplemental information. All input files including prior probability matrices, landmark files, and timescaled trees, output files from AncThresh and model comparison results are available online.

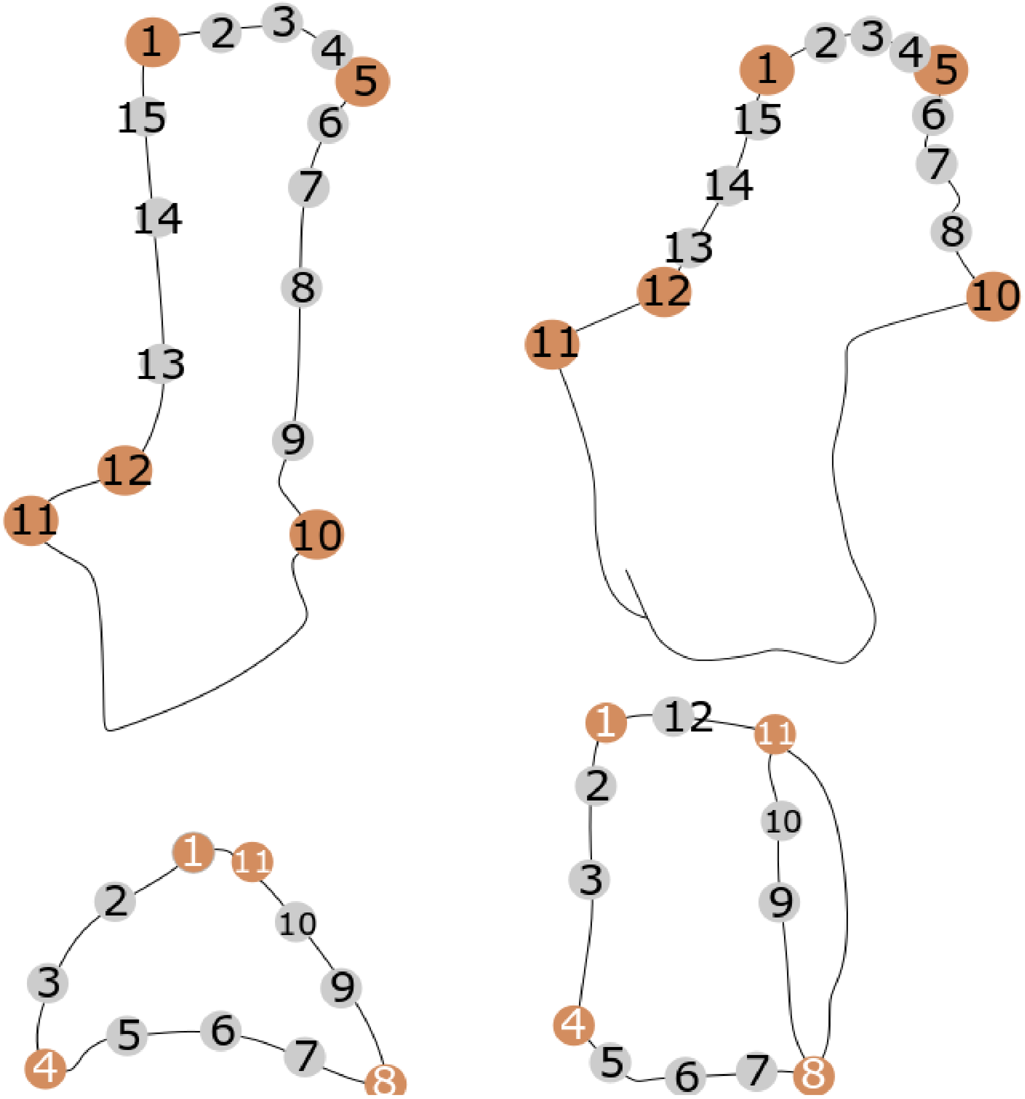

Fig. S1. Landmark schema on neural spines and intercentra of *Eryops* (left) and *Metoposaurus* (right). In gray type-II landmarks, in orange semi-sliding landmarks to generate curves

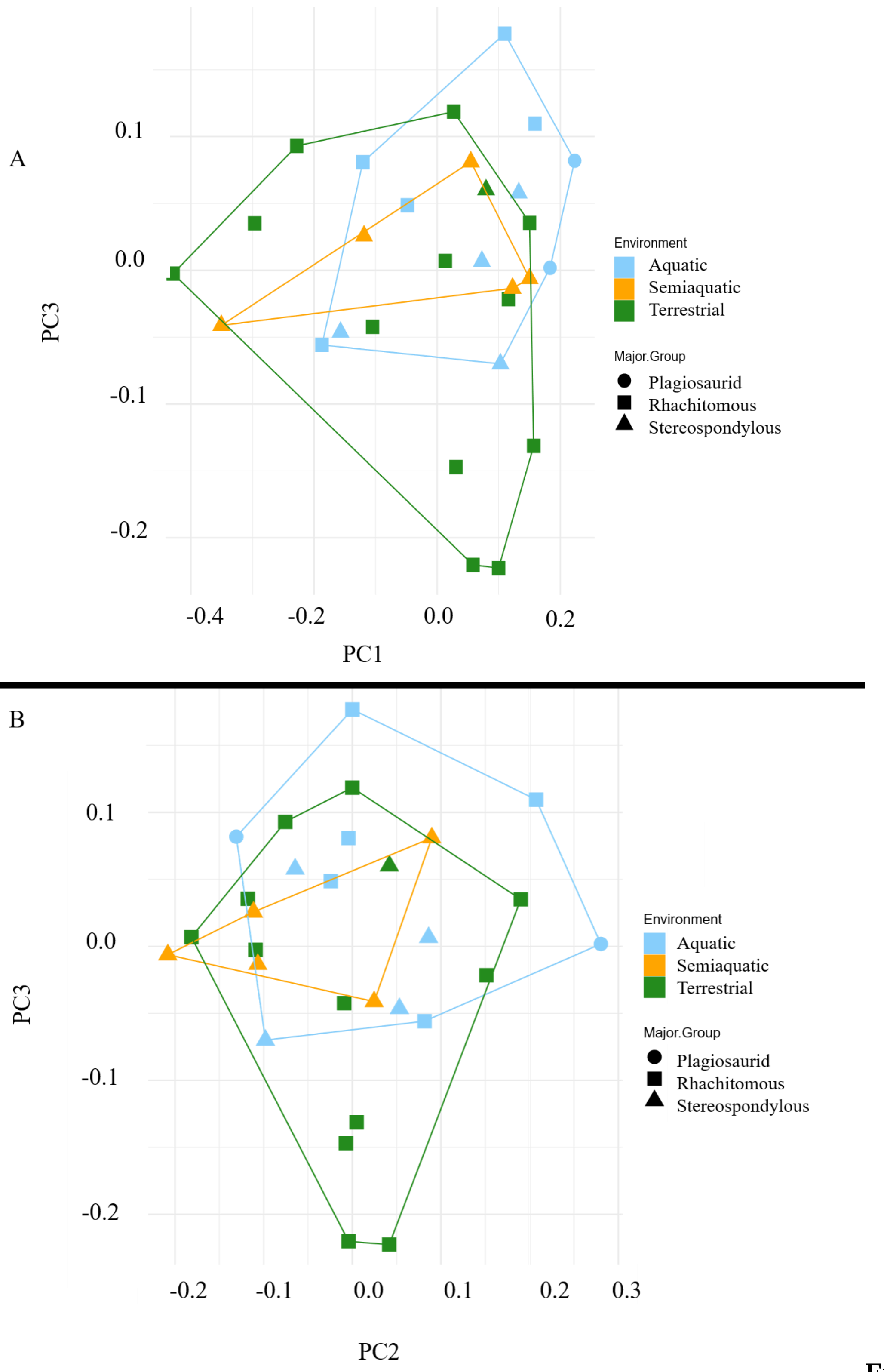

Fig.

Fig S2. Neural Spine morphospaces for temnospondyls. Principal component analysis for PCA 1v3 (A), and 2v3 (B).

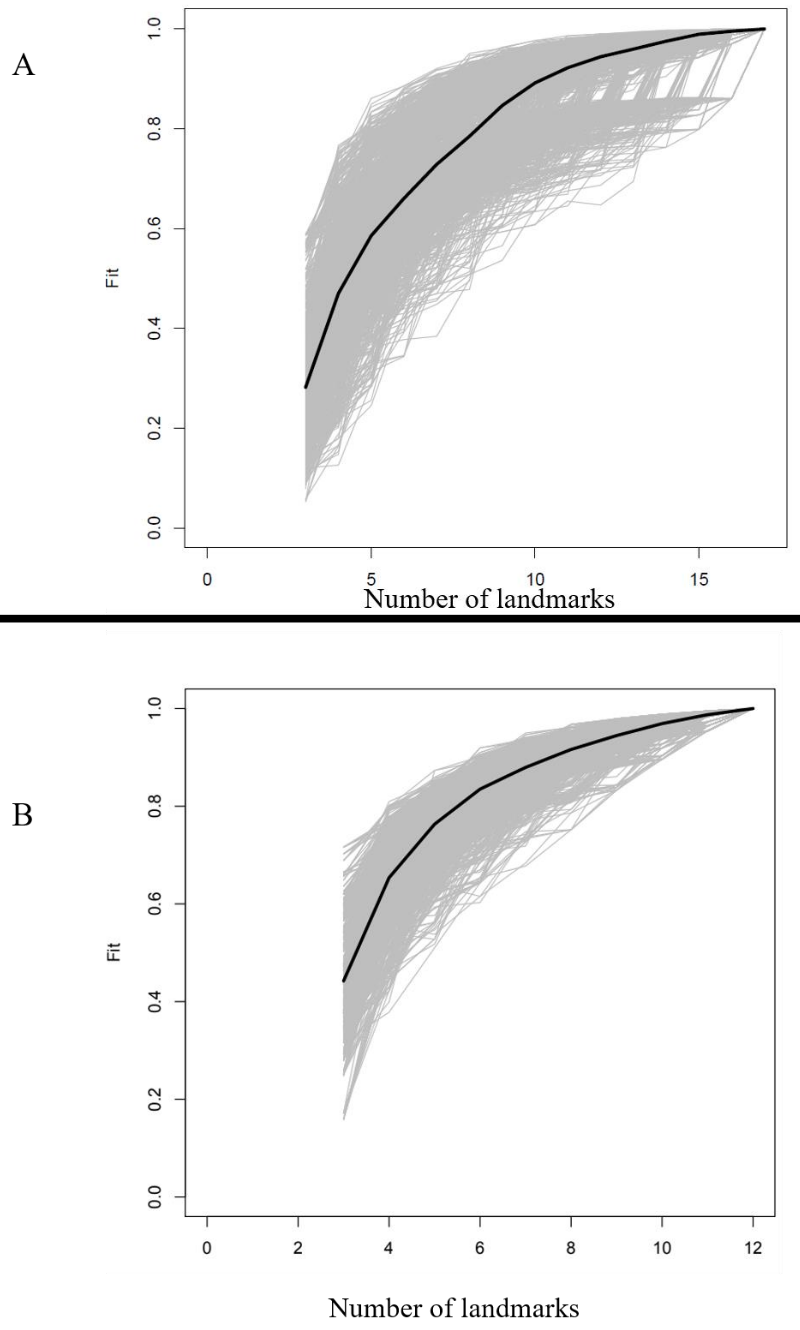

Fig. S3. Landmark sampling curves for the neural arches (A) and the intercentra (B). The plateaus indicate sufficient landmarking sampling.

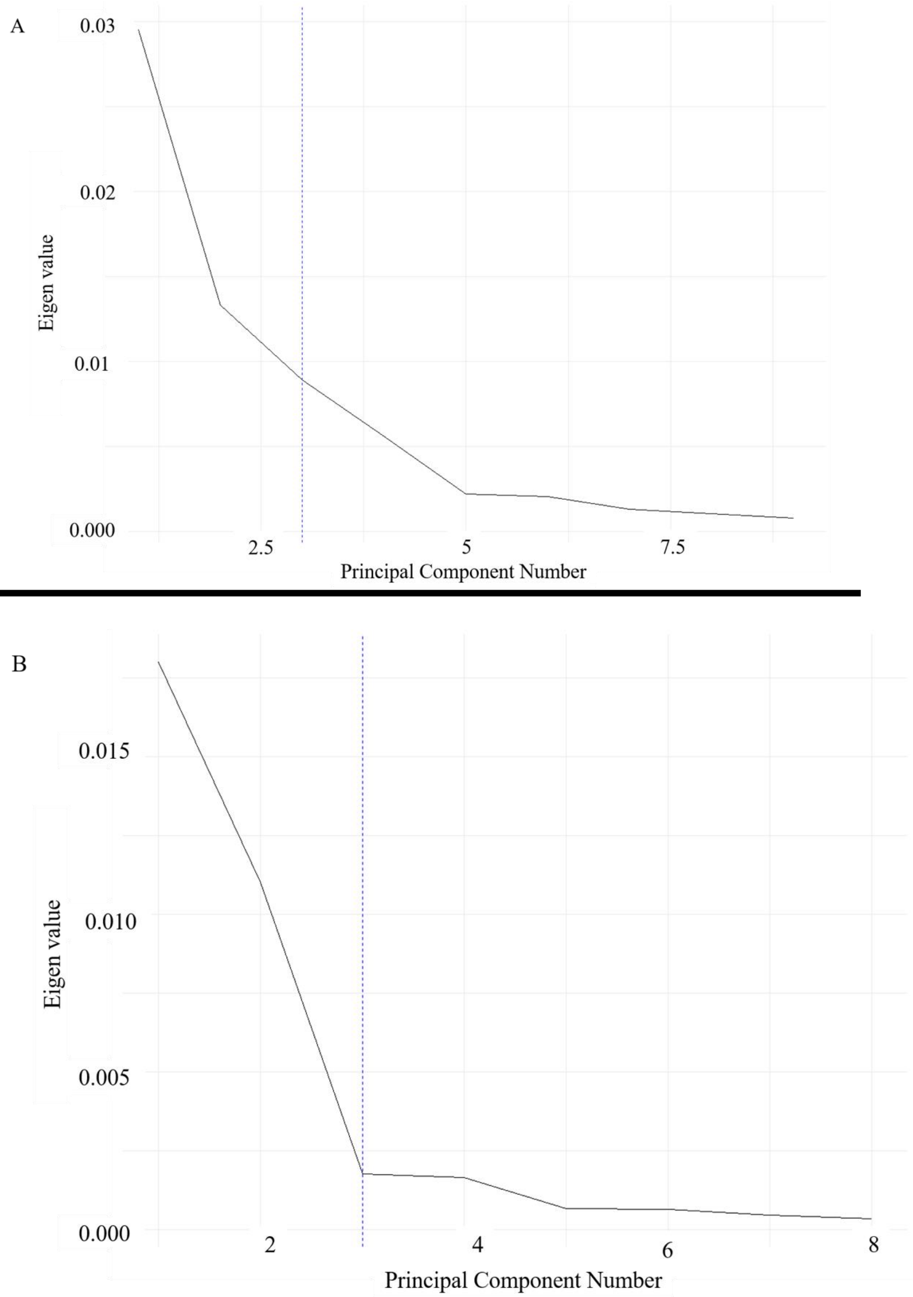

Fig. S4. The principal component number and eigen values for both the neural arches (A) and the intercentra (B). A drop off in eigen values is indicative of nonsignificant principal component analyses. Both intercentra and neural arches have a drop off near PC 3

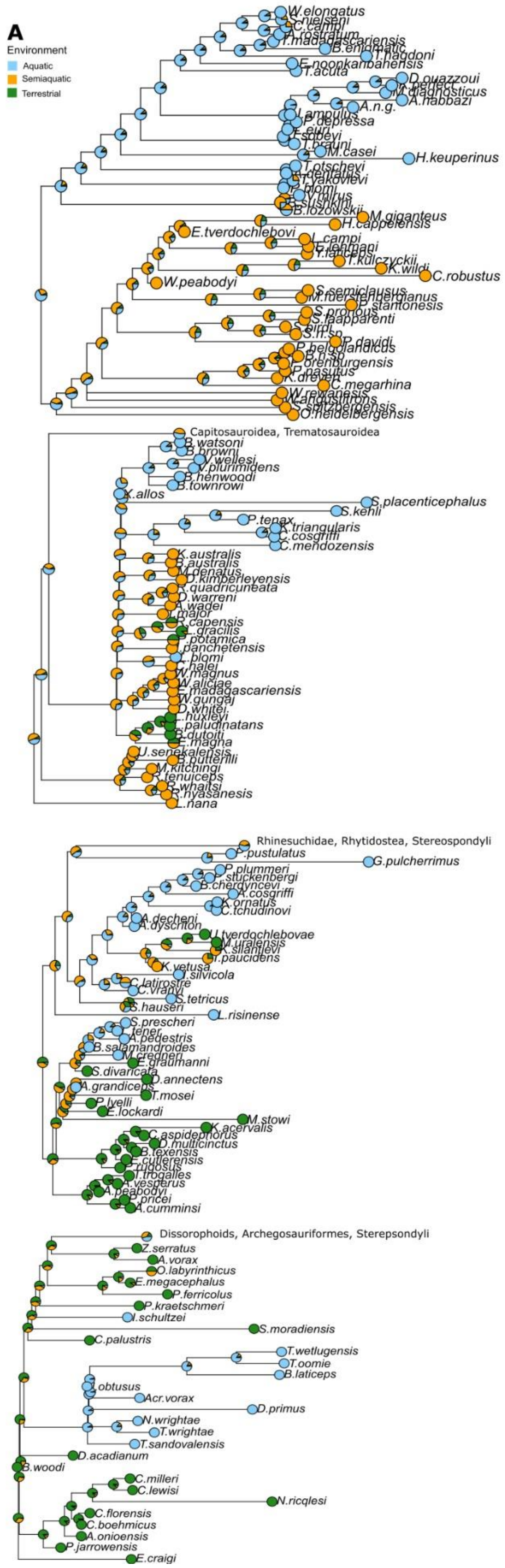
A
Environment
Aquatic
Semiaquatic
Terrestrial
Capitosauroidea, Trematosauroidea
Rhinesuchidae, Rhytidostea, Stereospondyli
Dissorophoids, Archegosauriformes, Sterepsondyli

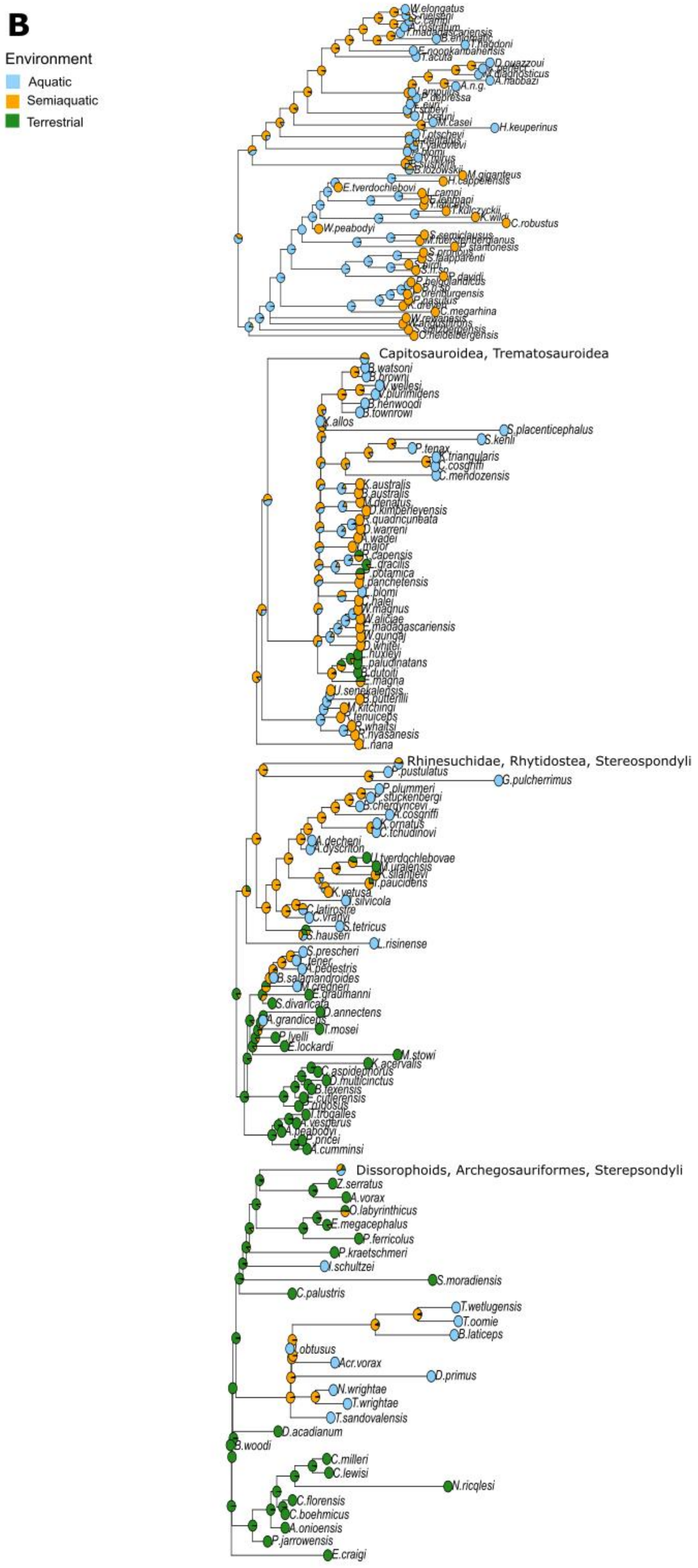
B
Environment
Aquatic
Semiaquatic
Terrestrial
Capitosauroidea, Trematosauroidea
Rhinesuchidae, Rhytidostea, Stereospondyli
Dissorophoids, Archegosauriformes, Sterepsondyli

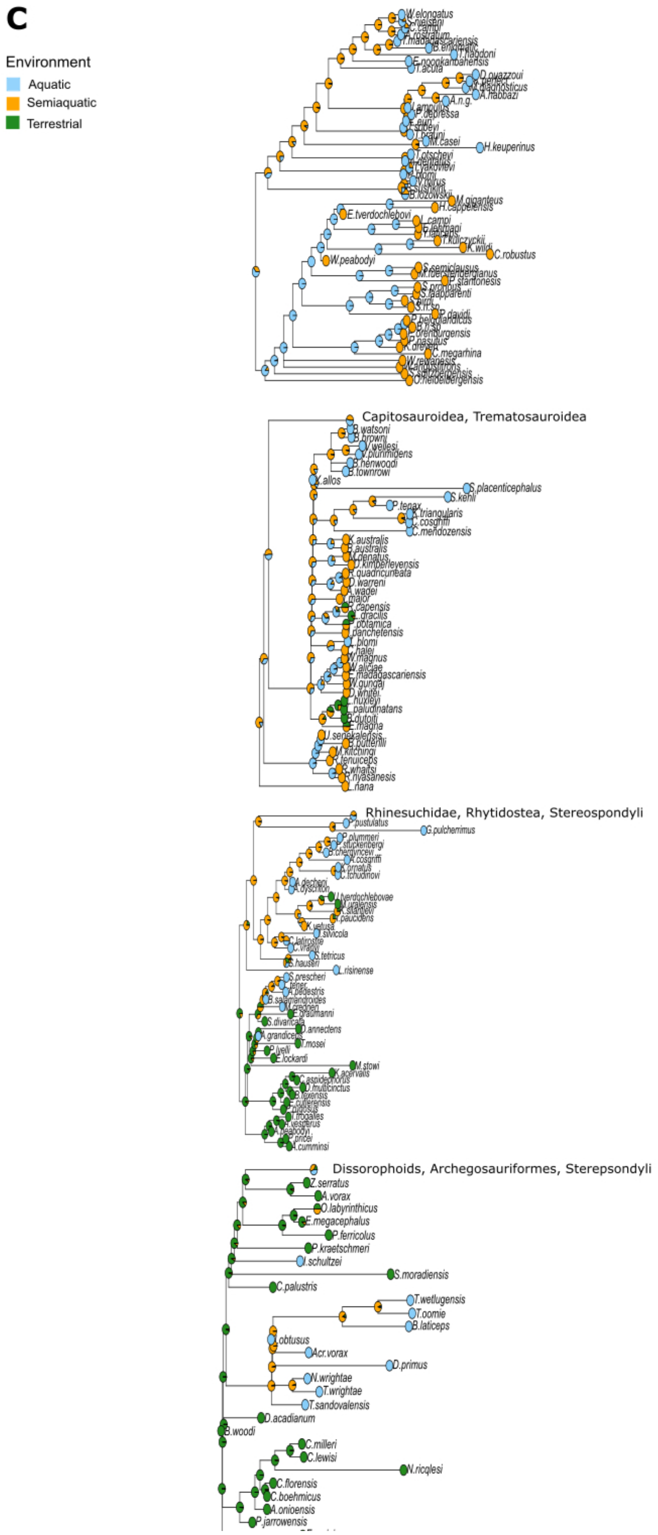

Fig. S5. Full Temnospondyl trees for the 3 models used in *AncThresh:* A) OU; B) Brownian; C) Lambda. Trees are split for formatting. Full results and trees are available in Dropbox file as cited in methods.

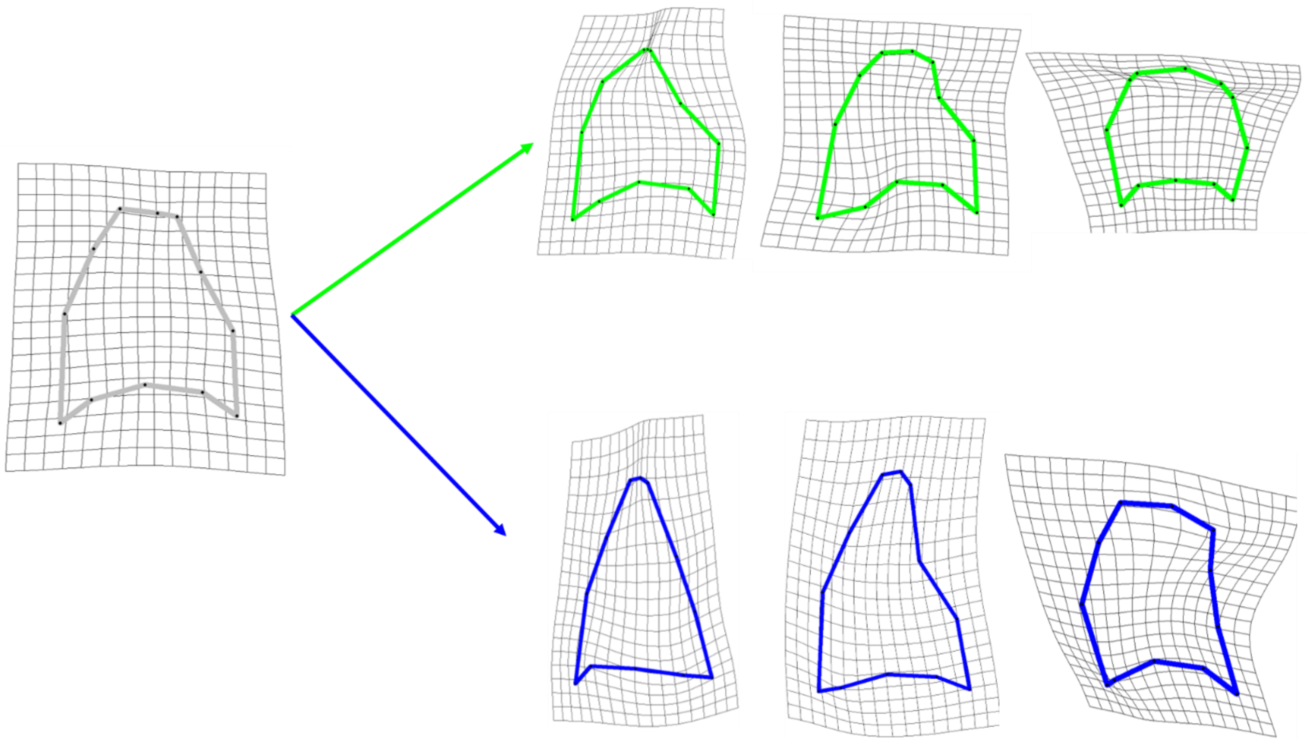

Fig. S6 Shape deformation grids of rhachitomous terrestrial intercentra taxa (top row, green) and aquatic taxa (bottom row, blue) from the consensus rhachitomous form (gray). There is a large diversity of shape among vertebrae all labeled rhachitomous.

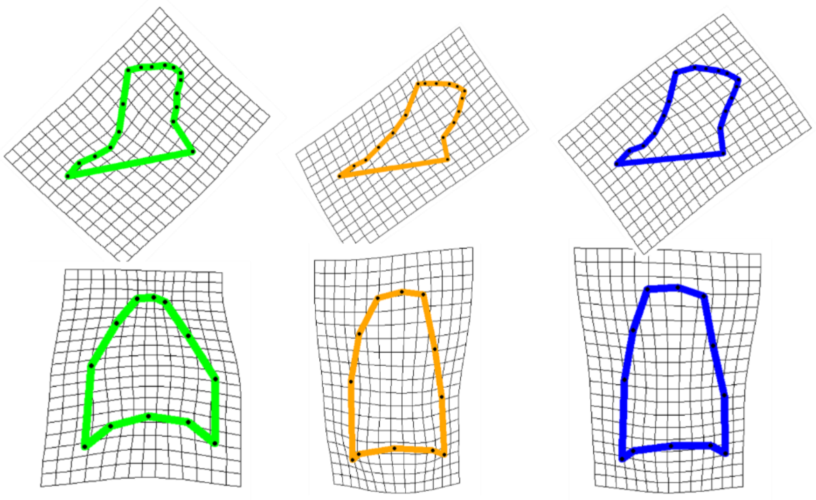

Fig. S7. Consensus morphologies of both neural arches (first row) and intercentra (second row) of each environmental group. Green = terrestrial, orange = semiaquatic, blue = aquatic. Neural arches had no environmental correlation and their shapes look very similar. Intercentra were correlated with environment and show a progression from terrestrial to aquatic forms

Table S1. Landmarking scheme for temnospondyl vertebral elements.

ELEMENT	LANDMARK	DESCRIPTION
INTERCENTRA	1	Cranial tip on the dorsal surface
	2:3	Semi-sliding landmark between landmarks 1 and 4
	4	Cranial tip on the ventral surface
	5:7	Semi-sliding landmark between landmarks 4 and 8
	8	Caudal tip on the ventral surface
	9:10	Semi-sliding landmarks between landmarks 8 and 11
NEURAL SPINE	1	Cranio-dorsal tip of the neural blade
	2:4	Sliding landmarks between cranio-dorsal and caudo-dorsal most tip of the neural lade
	5	Caudo-dorsal tip of the neural blade
	6:10	Semi-sliding landmarks between the caudo-dorsal tip of the neural blade to the caudal tip of the postzygapophysis
	11	Caudal tip of postzygapophysis
	12	Cranio-ventral tip of prezygapophysis
	13	Cranio-dorsal tip of prezygapophysis
	14:17	Semi-sliding landmark between cranio-dorsal tip of prezygapophysis and cranio-dorsal most point of the neural blade

Table S2. Species with both neural spines and intercentra, sample location, a priori environments (most recent environmental classification in parentheses), morphology governing the environmental classification. Sample source is for both elements unless otherwise specified.

Species	**Adult Environment (Most Recent)**	**Vertebral Type**	**Location**		**Morphological Criteria**
Archegosaurus dechani	Aquatic (Aquatic)	Rhachitomous	Reconstruction (IC)	Museum Photo (NS)	Branchial teeth on gill arches [1]
Aspidosaurus glascocki	Terrestrial (Terrestrial)	Rhachitomous	Museum Photo	AMNH 23412	Hyperossified skull [2]
Acanthostomatops vorax	Terrestrial (Terrestrial)	Rhachitomous	Reconstruction[3]	Figure 6	Robust limbs, lack of lateral lines, strong attachment of ischium to ilium [3]
Broiliellus novoamericanus	Terrestrial (Terrestrial)	Rhachitomous	Museum Photo	FMNH 1041	Robust limbs, lack of lateral lines[4]
Cacops aspidephorus	Terrestrial (Terrestrial)	Rhachitomous	Drawing[5]	Figure 1	Tympanic membranes specialized for airborne sound, well ossified appendicular skeleton, skull[6]
Dendrepeton acadium	Terrestrial (Terrestrial)	Rhachitomous	Reconstruction [5]	Figure 6	Tympanic membranes specialized for airborne sound[7]
Dissorophus multicinctus	Terrestrial (Terrestrial)	Rhachitomous	Museum photo	USNM 15555	Robust and well-ossified skeleton[5]
Doleserpeton annectens	Terrestrial (Terrestrial)	Rhachitomous	Drawing [8]	Figure 1	well-ossified appendicular skeleton, lacks lateral line[8]

Eryops megacephalus	Terrestrial (Terrestrial)	Rhachitomous	Museum Photo	AMNH 23565(IC) FMNH 745 (NS)	Biting stresses along the skull suggest terrestrial feeding[9]
Koskinonodon perfecta	Semiaquatic<br><br>(Aquatic)	Stereospondylous	Museum Photo	YPM 60249	Biting stresses along the skull suggest aquatic feeding, in addition to less ossified skeleton as compared to terrestrial taxa [10]
Lydekkerina huxleyi	Terrestrial (Terrestrial)	Stereospondylous	Reconstruction [11]	Figure 5	A well-defined medullary cavity, lack of calcified cartilage in long bone diaphysis, robust adductor crest for muscle attachment typical of terrestrial tetrapods[12]
Metoposaurus giganteus	Semiaquatic (Aquatic)	Stereospondylous	Museum photo	AMNH 3097	Presence of lateral line and histology of limb bones [13]
Mastodonosaurus diagnosticus	Aquatic (Aquatic)	Stereospondylous	Drawing[14]	Figure 28 (IC) Figure 33 (NS)	lateral lines, poorly appendicular skeleton, biting stresses along the skull suggest aquatic feeding[10]
Paracyclotosaurus davidi	Semiaquatic (Semiaquatic)	Stereospondylous	Drawing [15]	Figure 29	Humeral torsion high relative bone wall thickness[16]
Parioxys bolli	Terrestrial (Terrestrial)	Rhachitomous	Reconstruction [17]	Figure 1	Well ossified postaxial skeleton[18]

Phonerpeton pricei	Terrestrial (Terrestrial)	Rhachitomous	Reconstruction [19]	Figure 8	Skull sutures suggest terrestrial feeding[20]
Platyhystrix rugosus	Terrestrial (Terrestrial)	Rhachitomous	Museum Photo	UCMP 33437	Well ossified postaxial skeleton[21]
Neldasaurus wrightae	Aquatic (Aquatic)	Rhachitomous	Drawing [22]	Figure 8	Poorly ossified postaxial skeleton, enlarged interclavicle[23]
Sclerocephalus hauseri	Semiaquatic (Semiaquatic)	Stereospondylous	Drawing [24]	Figure 1	Lateral line, poorly ossified appendicular skeleton[25]
Siderops kehli	Aquatic (Semiaquatic)	Rhachitomous	Reconstruction [26]	Figure 1	Lateral line and laterally compressed tail[27]
Trimerorhachis insignis	Aquatic (Aquatic)	Rhachitomous	Museum Photo	UCMP 105157	Poorly ossified postaxial skeleton, enlarged interclavicle[23]

Table S3. species with neural spines only, sample location, and a priori environments

**Species**	**Environment**	**Vertebral Type**	**Location**		
Australerpeton cosgriffi	Aquatic (Aquatic)	Stereospondylous	Reconstruction [26]	Figure 1	long-snouted cranial morphology [28]
Acerastea wadei	Aquatic (Aquatic)	Stereospondylous	Drawing [26]	Figure 1	Poorly ossified appendicular skeleton [26]
Nooxobeia gracilis	Terrestrial (Terrestrial)	Rhachitomous	Reconstruction[29]	Figure 3	Well ossified postaxial skeleton[30]
Glaukerpeton avinoffi	Aquatic (Aquatic)	Rhachitomous	Literature Photograph[31]	Figure 15	Lateral line present [31]
Isodectes obtusus	Aquatic (Aquatic)	Rhachitomous	Drawing[32]	Figure 4	Preserved gills[33]
Parotosuchus pronus	Semiaquatic (Semiaquatic)	Stereospondylous	Drawing [26]	Figure 1	Biting stresses along the skull suggest amphibious feeding[34]
Plagiosuchus pustuliferus	Aquatic (Aquatic)	Plagiosaurid	Drawing[35]	Figure 1	Lateral line, ossified hyobranchium[36]

Table S4 **species with intercentra only, sample location, and a priori environments**

**Species**	**Environment**	**Vertebral Type**	**Location**		**Morphological criteria**
Batrchosuchus browni	Aquatic (Aquatic)	Stereospondylous	Museum Photo	UCMP 42856	Extensive latera canal system [37]
Bothriceps australis	Semiaquatic	Stereospondylous	Literature Photograph[38]	Figure 7	Poorly ossified postaxial skeleton [37]
Cylcotosaurus roboustus	Semiaquatic (Aquatic)	Stereospondylous	Museum Photo	UCMP V3957	Biting stresses along the skull suggest aquatic feeding[9]
Edops craigi	Terrestrial (Terrestrial)	Rhachitomous	Drawing[39]	Figure 11	Biting stresses along the skull suggest terrestrial feeding[9]
Laidleria gracilis	Terrestrial (Terrestrial)	Plagiosaurid	Reconstruction[40]	Figure 5	Well ossified postaxial skeleton[40]
Wellesaurus peabodyi	Semiaquatic (Aquatic)	Stereospondylous	Museum Photo	UCMP 56110	scalation patter suggests decreased need for belly protection and i turn primarily aquatic habitat[41]
Plagiobatrachus australis	Aquatic (Aquatic)	Stereospondylous	Literature Photograph[42]	Figure 3	Presence of diplospondyli [43]

Stenotosaurus semicalusus	Semiaquatic (Aquatic)	Stereospondylous	Museum Photo	UCMP 56108	Continuous lateral line grooves, limbs poorly ossified and reduced in size as compared to terrestrial taxa [43]
Tupilakosaurus heilmani	Aquatic (Aquatic)	Plagiosaurid	Reconstruction[44]	Figure 3	Number of diplospondylous vertebrae[44]
Thanbanchuia oomie	Aquatic (Aquatic)	Rhachitomous	Museum Photo	FMNH 1029/5	Ossified certaobranchials reduced limb ossifications, diplospondylous vertebrae[42]
Tupilakosaurus wetlugensis	Aquatic (Aquatic)	Plagiosaurid	Reconstruction[44]	Figure 3	Number of diplospondylous vertebrae[44]

Table S5. Principal component results from neural spines.

PRINCIPAL COMPONENT	EIGENVALUE	PROPORTION OF VARIANCE	CUMULATIVE PROPORTION
PC1	0.1642	0.4294	0.4294
PC2	0.1109	0.1956	0.625
PC3	0.09278	0.13702	0.76204
PC4	0.07135	0.08102	0.84308
PC5	0.04738	0.03574	0.87881
PC6	0.04317	0.02966	0.90848
PC7	0.03525	0.01978	0.92826
PC8	0.03177	0.01608	0.94432
PC9	0.02625	0.01097	0.95529
PC10	0.02378	0.009	0.96429
PC11	0.02065	0.00679	0.97108
PC12	0.01984	0.00627	0.97735
PC13	0.01765	0.00496	0.98231
PC14	0.01411	0.00317	0.98547
PC15	0.01373	0.003	0.98847
PC16	0.01263	0.00254	0.99101
PC17	0.01131	0.00204	0.99305
PC18	0.01105	0.00194	0.99499
PC19	0.008868	0.00125	0.994624
PC20	0.0086	0.00118	0.99742
PC21	0.007321	0.00085	0.99827
PC22	0.005953	0.00056	0.99884
PC23	0.004555	0.00033	0.9917
PC24	0.003885	0.00024	0.99941
PC25	0.003774	0.00023	0.99941
PC26	0.003488	0.00019	0.99983
PC27	0.002427	0.00009	0.99992

PC28	0.001799	0.00005	0.99997
PC29	0.001124	0.00002	0.99999

Table S6. Principal component summary results from intercentra.

PRINCIPAL COMPONENT	EIGENVALUE	PROPORTION OF VARIANCE	CUMULATIVE PROPORTION
PC1	0.194	0.694	0.6492
PC2	0.108	0.215	0.9093
PC3	0.048	0.043	0.9525
PC4	0.034	0.022	0.9748
PC5	0.0286	0.01513	0.9899
PC6	0.01672	0.00515	0.9951
PC7	0.01420	0.00371	0.9988
PC8	0.00655	0.000790	0.9996
PC9	0.00389	0.000280	0.9999
PC10	0.000219	0.000090	1.00

Table S7. ANOVA results for analyses of temnospondyl intercentra and neural spine shape on centroid size, habitat, vertebral type, and geologic era. Significant correlations are marked with an asterisk. Df = degrees of freedom, F = F-value, P = P=value.

	**Intercentra**				**Neural Spine**			
	Df	F	P	R^2	Df	F	P	R^2
Centroid Size	1	1.0006	0.368	0.032	1	1.351	0.195	0.047
Habitat	2	7.3734	0.001*	0.337	2	0.8155	0.583	0.059
Vertebral type	2	2.9985	0.001*	0.171	2	1.4725	0.148	0.102
Geologic Age	5	1.7648	0.036*	0.246	6	1.165	0.288	0.2412
Image Source	3	1.0273	0.404	0.099	3	1.300	0.209	0.135

Table S8. Phylogenetic least squares regression results for temnospondyl intercentra and neural spines.

	**Intercentra**				**Neural Spine**			
	Df	F	P	R^2	Df	F	P	R^2
Centroid Size	1	0.6341	0.656	0.0207	1	0.5473	0.554	0.198
Habitat	2	3.5434	0.002*	0.1963	2	0.4862	0.876	0.036
Vertebral type	2	2.0935	0.033*	0.1262	2	0.6945	0.626	0.051
Geologic Age	5	0.7722	0.758	0.1293	6	0.4522	0.952	0.110
Image Source	3	1.4422	0.073	0.1528	3	1.395	0.205	0.144

Table S9. Morphological disparity for temnospondyl intercentra. Pairwise differences are measured in Procrustes distances

	Aquatic		Semiaquatic		Terrestrial	
	Pairwise difference	P-value	Pairwise difference	P-value	Pairwise difference	P-value
Aquatic	0.000	1.00	0.00068	0.932	0.00016	0.979
Semiaquatic	0.00068	0.932	0.000	1.00	0.00052	0.946
Terrestrial	0.00016	0.979	0.00052	0.946	0.000	1.00

Table S10. Morphological disparity for temnospondyl neural spines. Pairwise differences are measured in Procrustes distances

	Aquatic		Semiaquatic		Terrestrial	
	Pairwise difference	P-value	Pairwise difference	P-value	Pairwise difference	P-value
Aquatic	0.000	1.00	0.0046	0.876	0.00998	0.633
Semiaquatic	0.0046	0.876	0.000	1.00	0.00529	0.823
Terrestrial	0.0099	0.633	0.0052	0.823	0.000	1.00

Table S11. Model Parameters from Ornstein-Uhlenbeck*(OU)*, Brownian(BM), Lambda (LB) *in AncThresh*. Starred order was discussed in manuscript. Inf = Infinite.

**Transition Order**	**Model**	**Mean Threshold Liabilities (10 mil. Gen. 10%burn-in)**			**Log Likliehood**	***Alpha***	**Phylogenetic Half-life (My)**
		**Terrestrial**	**Aquatic**	**Semiaquatic**			
Terrestrial – Semiaquatic – Aquatic*	OU	0	Inf	3.33	-624.306	0.26	2.65
	BM	0	Inf	3.51	-143.951	N/A	N/A
	LB	0	Inf	6.618	-799.515	0.98	0.705
Aquatic – Semiaquatic – Terrestrial	OU	Inf	0	3.892	-647.996	0.15	4.381
	BM	Inf	0	6.65	-783.624	N/A	N/A
	LB	Inf	0	6.811	-804.53	0.91826	0.705
Terrestrial – Aquatic - Semiaquatic	OU	0	0.904	Inf	-398.901	0.705	0.98
	BM	0	9.62	Inf	-790.274	N/A	N/A
	LB	0	10.63	Inf	-805.98	0.99	0.695

Table S12. Ornstein-Uhlenbeck (OU); Brownian (BM); Lambda(LB). Starred order was discussed in manuscript.

Transition Order	Model	DIC Value	DIC Weight
Terrestrial – Semiaquatic – Aquatic *	**BM**	1830	0
	**OU**	552	1
	**LB**	1861	0
Aquatic – Semiaquatic - Terrestrial	**BM**	1831	NaN
	**OU**	1690	NaN
	**LB**	1863	NaN
Terrestrial – Aquatic - Semiaquatic	**BM**	N/A	NaN
	**OU**	N/A	NaN
	**LB**	N/A	NaN

References

1. Witzmann F, Schoch RR. Skeletal development of the temnospondyl of *Acanthostomatops vorax* from the Lower Permian Döhlen Basin of Saxony. Earth and Environmental Science Transactions of The Royal Society of Edinburgh. 2005; 96: 365–385.

2. May W, Huttenlocker AK, Pardo JD, Benca J, Small BJ. New upper Pennsylvanian armored dissorophid records (Temnospondyli, Dissorophoidea) from the U.S. midcontinent and the stratigraphic distributions of dissorophids. Journal of Vertebrate Paleontology. 2011;31: 907–912.

3. Witzmann F, Schoch RR. Skeletal development of the temnospondyl *Acanthostomatops vorax* from the Lower Permian Döhlen Basin of Saxony. Trans Royal Society Edinburg: Earth Sciences. 2005;96: 365–385.

4. Holmes R, Berman DS, Anderson JS. A new dissorophid (Temnospondyli, Dissorophoidea) from the Early Permian of New Mexico (United States). Comptes Rendus Palevol. 2013;12: 419–435.

5. Dilkes D,W. Comparison and biomechanical interpretations of the vertebrae and osteoderms of *Cacops aspidephorus* and *Dissorophus multicinctus* (Temnospondyli, Dissorophidae). Journal of Vertebrate Paleontology. 2009; 29, 1013–1021.

6. Reisz R, Schoch R, Anderson J. The armoured dissorophid *Cacops* from the Early Permian of Oklahoma and the exploitation of the terrestrial realm by amphibians. Naturwissenschaften. 2009;96: 789.

7. Robinson J, Ahlberg PE, Koentges G. The braincase and middle ear region of *Dendrerpeton acadianum* (Tetrapoda: Temnospondyli). Zool J Linn Soc. 2005;143: 577–597.

8. Sigusen T, Bolt J.R. The Lower Permian amphibamid *Doleserpeton* (Temnospondyli: Dissorophoidea), the interrelationships of amphibamids, and the origin of modern amphibians. J Vertebr Paleontol. 2010; 30, 1360–1377.

9. Fortuny J, Marcé-Nogué J, De Esteban-Trivigno S, Gil L, Galobart À. Temnospondyli bite club: Ecomorphological patterns of the most diverse group of early tetrapods. J Evol Biol. 2011; 24(9): 2040-2054

10. Fortuny J, Marcé-Nogué J, Konietzko-Meier D. Feeding biomechanics of Late Triassic metoposaurids (Amphibia: Temnospondyli): a 3D finite element analysis approach. J Anat. 2017;230: 752–765.

11. Pawley K, Warren A. A terrestrial stereospondyl from the Lower Triassic of South

Africa: the postcranial skeleton of *Lydekkerina huxleyi* (Amphibia: Temnospondyli). Palaeontology.2005; 48(2): 281-298.

12. Canoville A, Chinsamy A. Bone Microstructure of the Stereospondyl *Lydekkerina huxleyi* Reveals Adaptive Strategies to the Harsh Post Permian-Extinction Environment. Anat Rec. 2015;298: 1237–1254.

13. Konietzko-Meier D, Sander PM. Long bone histology of *Metoposaurus diagnosticus* (Temnospondyli) from the Late Triassic of Krasiejów (Poland) and its paleobiological implications. J Vertebr Paleontol. 2013;33: 1003–1018.

14. Schoch RR. Comparative Osteology of Mastodonsaurus Giganteus (Jaeger, 1828) from the Middle Triassic (Lettenkeuper: Longobardian) of Germany (Baden-Württemberg, Bayern, Thüringen); with 4 Plates (Doctoral dissertation, Staatl. Museum für Naturkunde).1998.

15. Watson D.M.S. A new labyrinthodont *(Paracyclotosaurus*) from the upper Trias of New South Wales. Bulletin of the British Museum of Natural History, London (Geology). 1958; **3**, 233–263.

16. Mukherjee D, Sengupta DP, Rakshit N. New biological insights into the Middle Triassic capitosaurs from India as deduced from limb bone anatomy and histology. Pap Palaeontol. 2020;6: 93–142.

17. Carroll RL. The Relationships of the Rhachitomous Amphibian *Parioxy*s. American Museum Novitates. 1964; 2167.

18. DeMar R. The Permian labyrinthodont amphibian *Dissorophus multicinctus*, and adaptations and phylogeny of the family Dissorophidae. J Paleontol. 1968;42: 1210–1242.

19. Dilkes, D.W. A new trematopsid amphibian (Temnospondyli: Dissorophoidea) from the Lower Permian of Texas. Journal of Vertebrate Paleontology. 1990;*10*(2):222-243

20. Markey MJ, Marshall CR. Terrestrial-style feeding in a very early aquatic tetrapod is supported by evidence from experimental analysis of suture morphology. Proc Natl Acad Sci U S A. 2007;104: 7134–7138.

21. Schoch RR. Character distribution and phylogeny of the dissorophid temnospondyls. Fossil Record. 2012;15(2):121-37.

22. Chase JN. *Neldasaurus wrightae*: A New Rhachitomous Labyrinthodont from the Texas Lower Permian. Bulletin of the Museum of Comparative Zoology at Harvard College. 1965; 133: 153-225.

23. Pawley KA. The postcranial skeleton of *Trimerorhachis insignis* Cope, 1878 (Temnospondyli: Trimerorhachidae): a plesiomorphic temnospondyl from the Lower Permian of North America. Journal of Paleontology. 2007;81(5):873-94.

24. Schoch RR, Fastnacht M, Fichter J, Keller T. Anatomy and relationships of the Triassic temnospondyl *Sclerothorax*. Acta Palaeontologica Polonica. 2007;52(1).

25. Schoch RR. Life cycles, plasticity and palaeoecology in temnospondyl amphibians. Palaeontology. 2014;57(3):517-29.

26. Warren A, Snell N. The postcranial skeleton of Mesozoic temnospondyl amphibians: a review. Alcheringa. 1991 Jan 1;15(1):43-64.

27. Warren A, Damiani R. Stereospondyl Amphibians from the Elliot Formation of South Africa. Palaeontol Africana. 1999;54: 45–54.

28. Dias EV, Schultz CL. The first Paleozoic temnospondyl postcranial skeleton from South America. Revista brasileira de paleontologia. Rio de Janeiro, RJ. Vol. 6,(dez. 2003), p. 29-42. 2003.

29. Olson E. *Fayella chickashaensis*, the Dissorophoidea and the Permian terrestrial radiations. Journal of Paleontology.1972;1: 104-114

30. Gee BM, Scott D, Reisz RR. Reappraisal of the permian dissorophid *Fayella chickashaensis*. Can J Earth Sci. 2018;55: 1103–1114.

31. Werneburg R, Berman D. Revision of the Aquatic Eryopid Temnospondyl *Glaukerpeton avinoffi* Romer, 1952, from the Upper Pennsylvanian of North America. Annals of Carnegie Museum. 2012;81(1): pp.33-61.

32. Watson DM. The brachyopid labyrinthodonts. British Museum (Natural History); 1956.

33. Schoch RR, Witzmann F. Bystrow's Paradox–gills, fossils, and the fish-to-tetrapod transition. Acta Zoologica. 2011;92(3):251-65.

34. Fortuny J, Marcé-Nogué J, Gil L, Galobart À. Skull Mechanics and the Evolutionary Patterns of the Otic Notch Closure in Capitosaurs (Amphibia: Temnospondyli). Anat Rec. 2012;295: 1134–1146.

35. 14. Witzmann F, Soler-Gijón R. The bone histology of osteoderms in temnospondyl amphibians and in the chroniosuchian *Bystrowiella*. Acta Zoologica. 2010; 91: 96–114.

36. Konietzko-Meier D, Schmitt A. A histological study of a femur of *Plagiosuchus*, a Middle Triassic temnospondyl amphibian from southern Germany, using thin

sections and micro-CT scanning·. Netherlands Journal of Geosciences. 2013;92(2-3):97-108.

37. Warren A. Secondarily aquatic temnospondyls of the Upper Permian and Mesozoic. In Heatwole, H. & Carroll, RL (eds). Amphibian Biology, vol. 4: Palaeontology. Surrey Beatty, Chipping Norton: pp. 1121–1149.

38. Warren A, Rozefelds AC, Bull S. Tupilakosaur-like vertebrae in Bothriceps australis, an Australian brachyopid stereospondyl. Journal of Vertebrate Paleontology. 2011;31(4):738-53.

39. Romer AS, Witter RV. Edops, a primitive rhachitomous amphibian from the Texas red beds. The Journal of Geology. 1942;50(8):925-60.

40. 17. Warren A. *Laidleria* uncovered: a redescription of *Laidleria gracilis* Kitching (1957), a temnospondyl from the Cynognathus Zone of South Africa. Zoological Journal of the Linnean Society. 1998; 122 167–185

41. Witzmann F. The evolution of the scalation pattern in temnospondyl amphibians. Zoological Journal of the Linnean Society. 2007 Aug 1;150(4):815-34.

42. Warren A. Karoo tupilakosaurid: a relict from Gondwana. Earth and Environmental Science Transactions of The Royal Society of Edinburgh. 1998; 89, 145–160.

43. DeFauw S. Temnospondyl amphibians: a new perspective on the last phases in the evolution of the Labyrinthodontia. Mich Acad. 1989;21: 7–32.

44. Werneburg R, Steyer SJ, Sommer G, Gand G, Schneider JW, Vianey-Liaud M. The earliest tupilakosaurid amphibian with diplospondylous vertebrae from the Late Permian of southern France. Journal of Vertebrate Paleontology. 2007; 27: 26–30.
